# Cortical Motor Hierarchy Disruption Is Associated With Functional Status in Early Amyotrophic Lateral Sclerosis

**DOI:** 10.1002/jcsm.70411

**Published:** 2026-10-25

**Authors:** Jixin Luan, Yan Yun, Yichang Jiao, Yao Wang, Mingjie Ma, Didi Shan, Hongxu Wang, Xinbo Ji, Yao Tang, Jianing Li, Zexin Zhan, Xiaohan Sun, Ninglu Gao, Pengfei Lin, Chuanzhu Yan, Xiulian Sun, Dexin Yu, Shuangwu Liu, Fuchen Liu

**Affiliations:** ^1^ Department of Neurology, Research Institute of Neuromuscular and Neurodegenerative Diseases, Shandong Key Laboratory of Mitochondrial Medicine and Rare Diseases Qilu Hospital of Shandong University Jinan Shandong China; ^2^ Department of Radiology Qilu Hospital of Shandong University Jinan Shandong China; ^3^ Department of Radiology, Shandong Cancer Hospital and Institute Shandong First Medical University and Shandong Academy of Medical Sciences Jinan Shandong China; ^4^ School of Nursing and Rehabilitation, Cheeloo College of Medicine Shandong University Jinan Shandong China

**Keywords:** Allen Human Brain Atlas, amyotrophic lateral sclerosis, cortical functional gradients, imaging transcriptomics, muscle wasting, neurotransmitter

## Abstract

**Background:**

Amyotrophic lateral sclerosis (ALS) is a devastating neuromuscular disease characterised by progressive muscle wasting and functional decline. Although cortical motor network disruption is a hallmark of ALS, the large‐scale functional hierarchy alterations and their underlying molecular mechanisms in the early stage of the disease (ALS‐ES) remain poorly understood. We aimed to characterise cortical gradient changes in early‐stage ALS and to examine their neurochemical and transcriptomic correlates, as well as their cross‐sectional associations with clinical functional status.

**Methods:**

We recruited 97 patients with early‐stage ALS, all at King's stage 1 and 94 healthy controls with comparable age and sex distributions. The ALS‐ES group included 65 women and had a median ALSFRS‐R score of 40 (IQR, 37–44). Resting‐state fMRI was used to construct the principal functional gradient. We integrated neurochemical maps of 19 neurotransmitter receptors/transporters, and transcriptomic data from the Allen Human Brain Atlas. Partial least squares (PLS) regression and cell‐type enrichment analysis were employed to identify molecular correlates of gradient alterations.

**Results:**

Compared with HCs, ALS‐ES patients exhibited a significant spatial contraction of the principal gradient. Specifically, gradient values significantly increased in the somatomotor network (SMN; *t* = 3.670, *p*_FDR_ = 0.001) and decreased in the default mode network (DMN; *t* = −4.124, *p*_FDR_ < 0.001). Within the ALS‐ES group, higher SMN gradient values were associated with lower ALSFRS‐R scores, whereas higher DMN gradient values were associated with higher ALSFRS‐R scores. These macroscale alterations showed spatial correspondence with normative 5‐HT4 receptor and norepinephrine transporter maps. PLS analysis identified a primary gene set (PLS1+) enriched in oligodendrocytes and mitochondrial functions (*p*_FDR_ < 0.05), whereas the negative set (PLS1−) was associated with astrocytes, GABAergic neurons and RNA splicing pathways. Chromosome‐level analysis revealed significant enrichment of PLS1− genes on chromosomes 5 and 16 (*p* < 0.05).

**Conclusions:**

Early‐stage ALS is characterised by a collapsed cortical functional hierarchy centred on the motor‐somatosensory axis. These alterations were spatially associated with normative neurochemical maps and distinct transcriptomic annotations related to energy metabolism and RNA processing. These findings identify cross‐sectional cortical functional‐hierarchy alterations associated with functional status in early‐stage ALS and provide a multi‐scale framework for investigating their neurochemical and transcriptomic correlates.

## 1 Introduction

Amyotrophic lateral sclerosis (ALS) is currently considered a multisystemic disorder with widespread brain involvement [1]. Based on the new ‘Gold Coast’ criteria and the important conceptual framework of presymptomatic ALS, King's stage 1 patients are defined as early‐stage ALS (ALS‐ES) [2]. Uncovering the primary neurobiological alterations in this initial phase is essential to identify early molecular signatures, thereby facilitating early intervention strategies to delay disease prognosis. Although microscopic pathologies, such as TDP‐43 aggregation, are well characterised, systematically integrating these changes with macroscopic neuroimaging phenotypes remains a critical challenge [3, 4]. Consequently, it is crucial to explore abnormalities in the hierarchical organisation of brain function to determine whether these macroscopic disruptions reflect primary pathological mechanisms.

It is well established that ALS patients exhibit large‐scale brain network dysregulation, as evidenced by resting‐state functional magnetic resonance imaging (rs‐fMRI) studies. Specifically, changes in the functional connectivity (FC) patterns of the default mode network (DMN) and the sensorimotor network (SMN), which represent the opposing anchors of the cortical hierarchy, are closely associated with the core clinical symptoms of ALS [5]. Furthermore, connectome‐based imaging studies have found that abnormal FC in regions such as the prefrontal cortex, temporal lobe and motor cortex have been proposed as imaging features associated with ALS [6]. However, it remains unclear whether these changes in FC reflect a single, whole‐brain pattern of change, or if they present as multi‐level, heterogeneous spatial patterns within the hierarchical structure of the cortex. FC gradients, as an emerging imaging phenotype [7, 8], can effectively capture the hierarchical structure of functional integration in the brain, from primary sensory areas to high‐order association cortices, providing a new perspective for revealing the spatial heterogeneity of functional organisation in neuropsychiatric diseases [9, 10]. However, the underlying neurotransmitter, molecular and transcriptomic mechanisms of these gradients are still not well understood in ALS; a systematic link between changes in functional gradients and the distribution of neurotransmitters and gene expression patterns has yet to be established, particularly in ALS‐ES patients.

In recent years, the emergence of imaging‐transcriptomics has provided a new technical pathway for integrating macroscopic imaging phenotypes with microscopic gene expression information [11]. By combining neuroimaging phenotypes with high‐resolution brain region gene expression data, such as from the Allen Human Brain Atlas (AHBA), researchers can identify spatial gene expression patterns associated with specific imaging features and thereby uncover potential disease mechanisms [12]. In neurodegenerative disease research, imaging transcriptomics has been widely applied in fields such as Alzheimer's disease (AD) [13] and Parkinson's disease (PD) [14]. This approach helps researchers to systemically understand how genes shape the regional heterogeneity of brain structure and function, and it promotes the development of disease subtyping and precision intervention strategies. More importantly, imaging transcriptomics not only identifies gene sets related to imaging phenotypes but also uses multi‐platform integrated analysis to reveal the complex associations of these genes across multiple dimensions, including cell types, brain networks, cognitive functions and disease risk. For example, through cell‐type enrichment analysis, the gene sets linked by imaging‐transcriptomics can be mapped to specific cell types, thus elucidating their cell‐specific expression characteristics [13]. Simultaneously, using tools like Gene Annotation by Macroscale Brain‐imaging Association (GAMBA) and NeuroSynth [15], these gene sets can be further correlated with the topological properties of brain networks and cognitive functions, helping to reveal their potential roles in neural modulation processes [16]. Despite preliminary research suggesting that brain functional changes in patients with ALS may be related to specific gene expression profiles, the exact manner in which these genetic expression patterns are involved in ALS‐ES‐specific neuropathological processes still requires further in‐depth exploration.

This study integrated resting‐state fMRI gradients with normative neurotransmitter PET and AHBA transcriptomic data to characterise multiscale cortical alterations in ALS‐ES. We related regional gradient differences to neurotransmitter distributions and gene‐expression patterns using spatial correlation and partial least squares regression. Functional enrichment and cell‐type analyses were used to annotate the resulting gene sets. This framework provides hypothesis‐generating neurochemical and transcriptomic context for functional‐network disruption in ALS‐ES.

## 2 Materials and Methods

### 2.1 Study Participants

We included 97 newly diagnosed patients with ALS‐ES and 94 age‐ and sex‐matched healthy controls (HCs). All patients met the Gold Coast criteria for ALS and were classified as King's stage 1. Exclusion criteria included a family history of ALS, inability to undergo MRI or comorbidity of other neurological disorders. Clinical data were collected for all participants, including anxiety symptoms assessed using the Hamilton Anxiety Rating Scale (HARS) and depressive symptoms assessed using the Hamilton Depression Rating Scale (HDRS). Disease severity was assessed in patients with ALS‐ES using the Amyotrophic Lateral Sclerosis Functional Rating Scale‐Revised (ALSFRS‐R). The flowchart is shown in Figure 1. Full inclusion/exclusion criteria and all clinical assessments are detailed in the Supplementary Methods S1.

**FIGURE 1:**
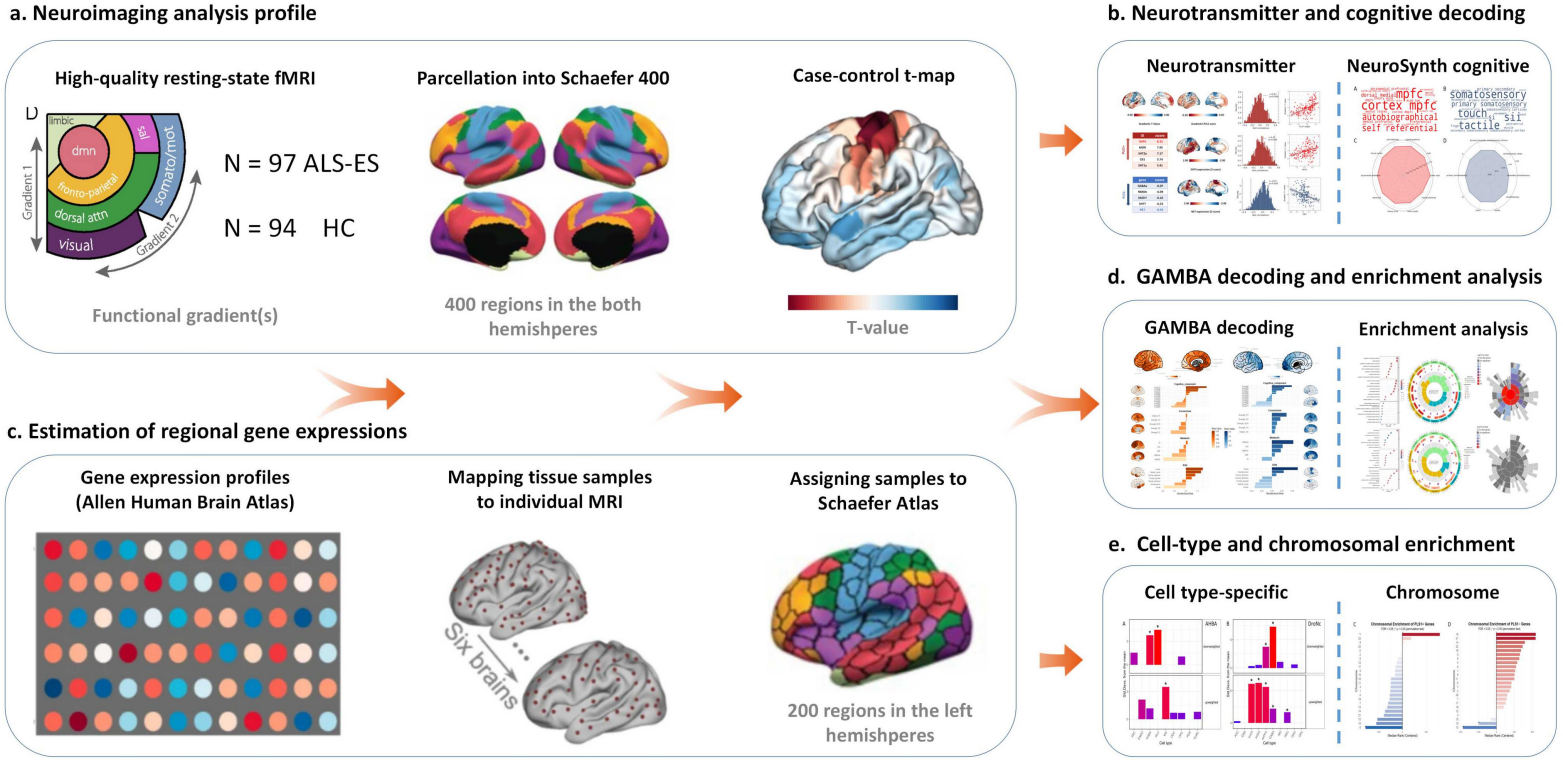
A flowchart of the study. (a) Neuroimaging analysis profile. Resting‐state fMRI data from 97 early‐stage ALS patients and 94 HCs were analysed, estimating first and secondary gradients at the voxel level using a general linear model to generate case–control t‐maps. (b) Neurotransmitter and cognitive decoding. The case–control t‐map was registered to the Schaefer‐400 atlas, with PLS regression linking t‐map values to neurotransmitter data. Cognitive relevance was decoded using NeuroSynth and visualized in a word cloud. (c) Estimation of regional gene expressions. Gene expression data from the Allen Human Brain Atlas were processed and PLS regression identified gradient‐associated gene‐expression patterns. (d) Gene Annotation by Macroscale Brain‐imaging Association (GAMBA) decoding and enrichment analysis. GAMBA was used to correlate transcriptional signatures with brain networks and disease phenotypes, followed by Gene Ontology (GO) and Kyoto Encyclopedia of Genes and Genomes (KEGG) pathway enrichment analysis. (e) Cell‐type and chromosomal enrichment. Cell type‐specific and chromosomal enrichment analyses were performed for the identified gene sets. ALS‐ES, early‐stage amyotrophic lateral sclerosis; HCs, healthy controls.

### 2.2 MRI Examination and Data Preprocessing

All MRI data were acquired using a 3.0‐T magnetic resonance system (Philips Medical System Ingenia scanner) equipped with a dStream head coil. During the scan, all participants were instructed to remain still and lie supine. All structural and resting‐state functional MRI data were converted to BIDS format and preprocessed using fMRIPrep. The T1‐weighted images underwent skull‐stripping, brain tissue segmentation and surface reconstruction. Functional data were processed through head‐motion correction, co‐registration to the structural image and surface‐based resampling. Nuisance signal regression was performed using a comprehensive set of 36 regressors. The preprocessed time series were then band‐pass filtered (0.01–0.1 Hz) and smoothed with a 6‐mm full width at half maximum (FWHM) kernel on the cortical surface. Full details for the process of MRI acquisition and the image preprocessing pipeline can be found in Supplementary Method S2.

#### 2.2.1 Group Comparisons of Functional Gradients

We selected the first two gradient components for subsequent analysis. According to the Yeo7 brain network template, the 400 brain regions were assigned to seven distinct networks, allowing comparisons among the 400 cortical regions and large‐scale cortical networks. To evaluate group‐level differences in functional gradient patterns across brain regions and networks, we applied ordinary least squares (OLS) models, with age and sex included as covariates. Group comparisons included ALS‐ES versus HCs. We established statistical significance levels at *p* < 0.05, with multiple comparisons corrected using the Bonferroni false discovery rate method. Group‐level analyses were adjusted for age and sex. Additional clinical, effect‐size and sensitivity analyses, including adjustment for HARS and HDRS, sex‐stratified analyses and exclusion of patients with bulbar onset, are described in the Supplementary Methods S3.

### 2.3 Neurotransmitter and Cognitive Decoding of Gradient Alterations

To decode the neurobiological and cognitive relevance of case–control gradient alterations, we performed neurotransmitter‐receptor mapping and meta‐analytic cognitive decoding. Partial least squares regression was used to model the relationship between regional gradient differences and a multi‐receptor/transmitter positron emission tomography (PET) atlas (*n* = 1200). Additionally, spatial correlation analysis against the NeuroSynth database linked gradient changes to specific cognitive functions. In brief, we first converted case–control t‐map from surface space to volume space in BrainStat. Then, the spatial correlation was conducted between the t‐map and meta‐analytic map of each term from the NeuroSynth database. Full methodological details are provided in Supplementary Method S4.

### 2.4 Estimation of Regional Gene Expressions

Regional gene expression data were derived from the AHBA, including microarray data from six postmortem brains processed according to established protocols [17]. Expression values were mapped to 200 left‐hemisphere cortical regions. To identify transcriptomic correlates of regional gradient alterations, we applied PLS regression between case–control *t*‐values and genome‐wide expression profiles (15 633 genes). Significant genes (Bonferroni‐corrected *p* < 0.05) were identified via spherical permutation testing and bootstrap stability assessment. Genes with positive weights on the primary PLS component were designated as PLS1+, whereas those with negative weights were designated as PLS1−, collectively comprising the gradient‐associated gene sets. Full methodological details are provided in Supplementary Method S5.

#### 2.4.1 Transcriptomic and Neuropsychiatric Decoding via GAMBA

The GAMBA platform serves as a pathophysiological database for meta‐analytic gene‐list annotation, facilitating the decoding of gradient relationships and neuropsychiatric associations [18]. Genes identified through a prior PLS analysis and surviving Bonferroni method correction were bifurcated into positive (PLS1+) and negative (PLS1−) weight subsets. We employed a general linear regression model to associate these gene sets with various neuroimaging phenotypes (e.g., resting‐state networks, cortical metabolism). Subsequently, activation‐likelihood estimate (ALE) analysis was used to determine the neuropsychiatric and neurological associations of these gene sets. Specific statistical models and correction methods are described in Supplementary Methods S6.

### 2.5 Transcriptomic Functional Enrichment Analysis

To elucidate the biological relevance of the PLS1+ and PLS1− gene sets, we performed comprehensive bioinformatics analyses. These analyses encompassed Gene Ontology (GO) functional annotation, pathway enrichment via Metascape and synaptic functional annotation using SynGO. Furthermore, we evaluate the overlap between our gene sets and established ALS risk factors through integration of data from large‐scale genome‐wide association studies (GWAS), including a study of over 240 000 European individuals (accession: ebi‐a‐GCST90027164) and summary‐data‐based Mendelian randomisation (SMR) studies. Detailed information on all databases, software packages and statistical thresholds is provided in Supplementary Methods S7.

#### 2.5.1 Cell‐Type and Chromosome Enrichment Analysis

We applied expression‐weighted cell type enrichment (EWCE) to determine if the PLS1+ and PLS1− gene subsets were enriched in specific brain cell types (e.g., astrocytes, microglia, neurons). This analysis was performed on the AHBA‐seq single‐cell dataset [19] and independently validated using the DroNc‐seq dataset [20]. We also performed a chromosome enrichment analysis using a permutation‐based median rank method to test for non‐random clustering of genes on specific chromosomes. Full methodological details for both analyses are available in Supplementary Methods S8. Detailed statistical prioritisation criteria for distinguishing core, robust secondary and exploratory findings are provided in Supplementary Methods S9.

## 3 Results

### 3.1 Demographic Information and Clinical Characteristics

The demographic and clinical characteristics of patients with ALS‐ES (*n* = 97) and HCs (*n* = 94) are summarised in Table 1. There were no significant differences between the two groups in age (ALS‐ES: 57.12 ± 7.20 years; HCs: 56.14 ± 6.42 years; *p* = 0.320) or sex distribution (male/female: 32/65 vs. 35/59; *p* = 0.539). Among patients with ALS‐ES, 23 patients had bulbar onset, representing 23.7% of the cohort. Median disease duration was 15 months, with an interquartile range of 12 to 18 months, and the median ALSFRS‐R score was 40, with an interquartile range of 37 to 44. HARS and HDRS scores were higher in ALS‐ES patients than in HCs. Median HARS scores were 11 and 5, respectively, and median HDRS scores were 7 and 2, respectively. Mean framewise displacement did not differ significantly between ALS‐ES patients and HCs (0.237 ± 0.114 vs. 0.212 ± 0.094, respectively; *p* = 0.106).

**TABLE 1:** Clinical data of ALS‐ES and HCs.

Variable	ALS‐ES (*n* = 97)	HCs (*n* = 94)	*p*
Age (years)	57.12 ± 7.20	56.14 ± 6.42	0.320
Sex, *n* (%)			0.539
Male	32 (33.0)	35 (37.2)	
Female	65 (67.0)	59 (62.8)	
Bulbar ALS onset n, (%)	23 (23.7)	—	—
ALS duration (month)	15 (12, 18)	—	—
ALSFRS‐R scores	40 (37, 44)	—	—
HARS	11 (6, 14)	5 (2, 7)	< 0.01
HDRS	7 (3, 11)	2 (0, 4)	< 0.01
Mean FD	0.237 ± 0.114	0.212 ± 0.094	0.106

*Note:* Data are presented as mean ± standard deviation for normally distributed continuous variables, median (first quartile, third quartile) for non‐normally distributed continuous variables or *n* (%).

Abbreviations: ALS‐ES, early‐stage amyotrophic lateral sclerosis; ALSFRS‐R, Amyotrophic Lateral Sclerosis Functional Rating Scale‐Revised; FD, framewise displacement; HARS, Hamilton Anxiety Rating Scale; HCs, healthy controls; HDRS, Hamilton Depression Rating Scale.

### 3.2 Global Functional Gradient Features

We first characterised the global features of the first functional gradients. We focused on the first two functional gradients, which collectively explained over 10% of the total variance (Figure S1). Group‐level maps revealed the expected hierarchical organisation, with the first gradient spanning from unimodal to transmodal networks (Figure S2a) and the second gradient capturing the transition from visual/somatomotor cortices to the DMN (Figure S2c). Although this fundamental topology was preserved in ALS‐ES patients, we found a significant spatial contraction of the functional hierarchy (Figure S2d,e). This alteration was specific to the first functional gradient: ALS‐ES patients showed a significantly reduced gradient range (*p* = 0.020), standard deviation (*p* = 0.007) and variance ratio (*p* = 0.008). In contrast, all metrics for the second gradient were preserved (all *p* > 0.05), indicating no significant group differences (Table S1).

### 3.3 Regional and Network‐level Functional Gradients

We next examined regional and network‐level alterations. After controlling for age and sex, we found significant alterations specific to the first functional gradient in ALS‐ES patients. At the regional level, patients exhibited decreased gradient values in DMN nodes (e.g., prefrontal and temporal regions) and increased values in bilateral somatomotor network (SMN) regions (Table S2). These regional effects were mirrored at the network level: The DMN showed a significant reduction in gradient values (*t* = −4.124, *p*_FDR_ < 0.001), whereas the SMN exhibited a significant increase (*t* = 3.670, *p*_FDR_ = 0.001) (Figure 2; Table S3). In stark contrast, no significant differences were found for the second functional gradient at either the regional level (Table S4) or the network level after FDR correction. The overall distributions are visualised in ridge plots (Figure S3). Collectively, these findings point to a disrupted functional hierarchy in ALS‐ES, characterised by higher SMN and lower DMN Gradient‐1 values. Within the ALS‐ES group, higher SMN Gradient‐1 values were associated with lower ALSFRS‐R scores (*r* = −0.274, *p*_FDR_ = 0.015), whereas higher DMN values were associated with higher ALSFRS‐R scores (*r* = 0.344, *p*_FDR_ = 0.004) (Table S5). No group × sex interaction survived FDR correction across the seven canonical networks (Table S6). Standardised effect sizes for all seven networks are reported in Table S7. The primary SMN increase and DMN decrease remained significant after additional adjustment for mean framewise displacement, HARS and HDRS (Table S8). The primary SMN increase and DMN decrease were also retained after exclusion of patients with bulbar onset (Table S9).

**FIGURE 2:**
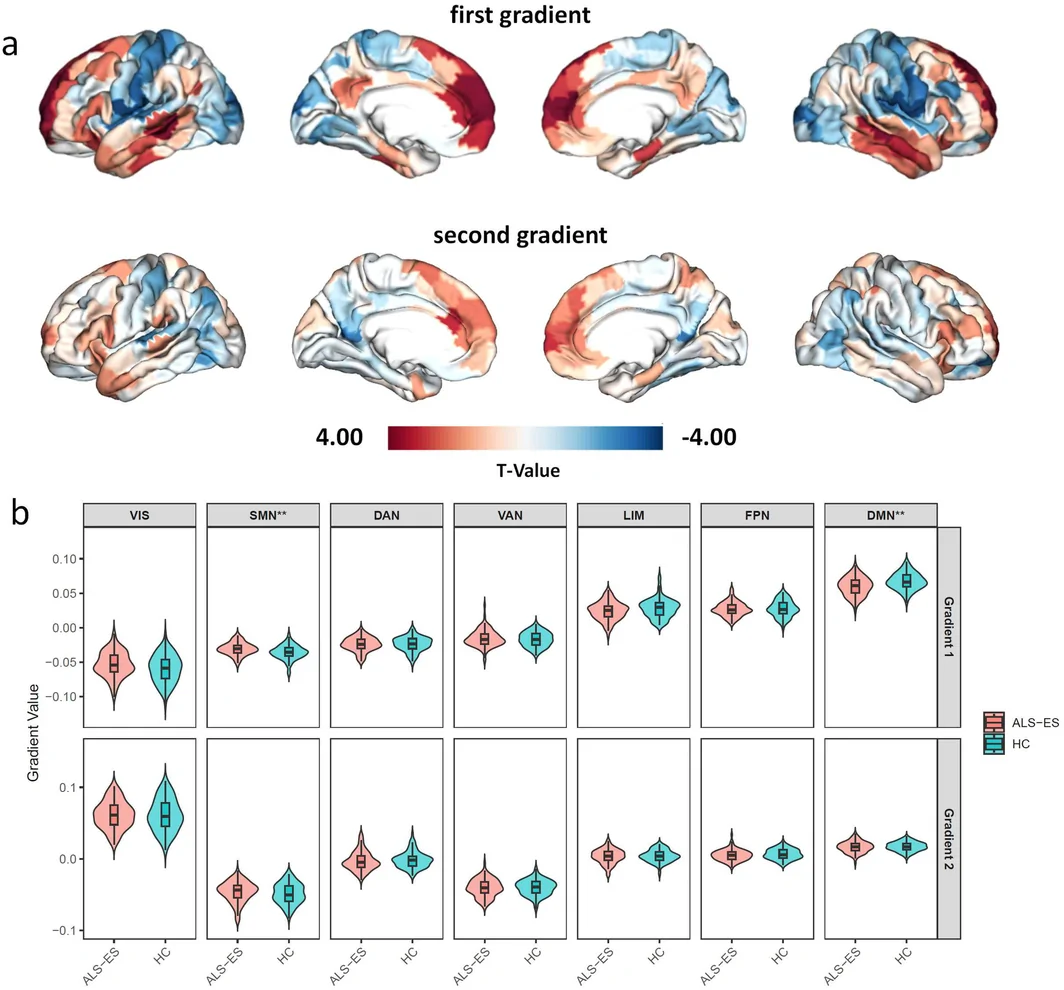
Group differences in functional gradients between ALS‐ES patients and HCs. (a) T‐statistic map showing spatial distribution of between‐group differences in the first and second functional gradients. Warm/cool colours represent increased/decreased values in patients. (b) Violin plots illustrating the distribution of the first and second functional gradients in each group. ALS‐ES, early‐stage amyotrophic lateral sclerosis; DAN, dorsal attention network; DMN, default mode network; FPN, frontoparietal network; HCs, healthy controls; LIM, limbic network; SMN, somatomotor network; VAN, ventral attention network; VIS, visual network.

### 3.4 Normative Neurochemical Correlates of Gradient‐1 Alterations

To characterise the spatial correspondence between regional Gradient‐1 alterations and normative neurochemical distributions, we applied PLS analysis linking the case–control Gradient‐1 alteration map to PET‐derived maps of 19 neurotransmitter receptors and transporters from healthy reference datasets (Figure 3a). The first PLS component (PLS1) accounted for 24.81% of the variance in cortical alterations (*p*_*boot*_ = 0.001), with its weighted neurotransmitter expression pattern showing a significant positive correlation with differences in the first gradient (*r* = 0.51, *p*_*spin*_ < 0.001) (Figure 3b,c). Nineteen neurochemical markers were identified in total. Seven PLS1‐positive markers (*Z* > 2.10, *p*_*FDR*_ < 0.05) were positively associated with increases in the first gradient, whereas six PLS1‐negative markers (*Z* < −2.10, *p*_*FDR*_ < 0.05) were negatively associated with its decreases (Table S10). The regional Gradient‐1 alteration map showed positive spatial correspondence with the normative 5‐HT4 receptor distribution and negative spatial correspondence with the normative norepinephrine transporter distribution. Notably, the serotonergic 5‐hydroxytryptamine receptor 4 (5‐HT4), strongly weighted on PLS1, exhibited a positive correlation with differences in the first gradient (*r* = 0.353, *p*_*spin*_ = 0.004) (Figure 3d). Conversely, noradrenaline transporters (NET), negatively weighted on PLS1, were inversely correlated with gradient changes (*r* = −0.314, *p*_*spin*_ = 0.004) (Figure 3e).

**FIGURE 3:**
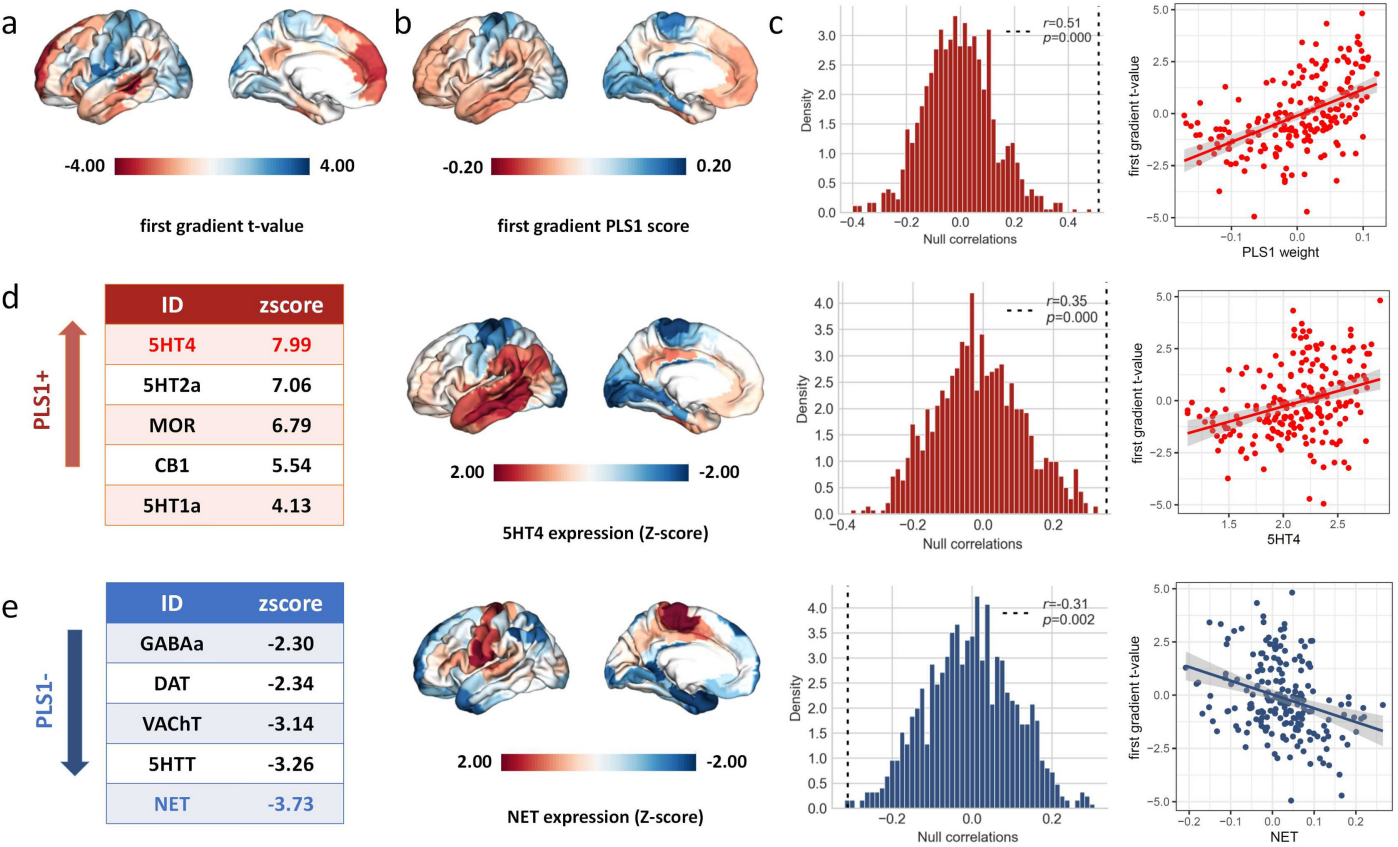
Normative neurochemical maps spatially associated with spatial changes in the first functional gradient. (a) T‐statistic map illustrating between‐group differences in the first functional gradient within the left cerebral hemisphere in ALS‐ES patients relative to HCs. (b) Spatial distribution of neurotransmitter expression weights based on PLS1 component scores in the left hemisphere. (c) Neurotransmitter expression pattern encoded by PLS1. Scatter plot demonstrates a significant positive correlation between regional PLS1 scores and *T*‐values of the first functional gradient differences (*r* = 0.51, *P*_spin_ < 0.001); red dots denote 200 regions of interest. (d) Neurotransmitters strongly positively weighted on PLS1 (e.g., 5‐HT4) are positively correlated with case–control differences in the first functional gradient (*r* = 0.35, *P*_spin_ < 0.001). (e) Neurotransmitters strongly negatively weighted on PLS1 (e.g., NET) are negatively correlated with case–control differences in the first functional gradient (*r* = −0.31, *P*_spin_ = 0.002). 5‐HT1a, 5‐hydroxytryptamine receptor 1A; 5‐HT2a, 5‐hydroxytryptamine receptor 2A; 5‐HT4, 5‐hydroxytryptamine receptor 4; 5‐HTT, 5‐hydroxytryptamine transporter; CB1, cannabinoid receptor 1; DAT, dopamine transporter; GABAa, gamma‐aminobutyric acid type A receptor; MOR, mu‐opioid receptor; NET, norepinephrine transporter; PLS, partial least squares; VAChT, vesicular acetylcholine transporter.

### 3.5 Association Between Cognitive Terms and ALS Alterations

To understand the functional relevance of these gradient alterations, we performed meta‐analytic cognitive decoding. We found that alterations in the first functional gradient were strongly aligned with distinct cognitive and functional domains (Figure S4). Specifically, gradient alterations were positively correlated with DMN‐related transmodal functions, including theory of mind (*r* = 0.36), medial prefrontal cortex (*r* = 0.36) and autobiographical memory (*r* = 0.32). Conversely, they showed strong negative correlations with unimodal sensorimotor functions, such as secondary somatosensory cortex (*r* = −0.45), somatosensory (*r* = −0.44) and tactile processing (*r* = −0.42) (Figure S4; Table S11). This strong functional‐spatial alignment was specific to the first gradient. A similar analysis of the second functional gradient revealed a similar pattern of associations but with substantially weaker correlation values. For example, the top positive correlation (mpfc) was only *r* = 0.31, and a key negative correlation (sensorimotor cortex) was only *r* = −0.14 (Figure S5; Table S11). These findings suggest that ALS‐related disruptions of the functional hierarchy are primarily and strongly associated with the transmodal (social cognition) and unimodal (sensorimotor) systems captured by the first functional gradient.

#### 3.5.1 Regional Gene Expression Pattern of Gradient Perturbation

To investigate the genetic basis of this reorganisation, we employed PLS regression to relate gene expression patterns to the anatomical distribution of the first functional gradient. This revealed a significant primary component (PLS1) that explained 23.50% of the gradient's variance (*p*_*boot*_ = 0.001). This PLS1‐weighted gene map was strongly correlated with the ALS‐related (case–control) t‐map of the first gradient (*r* = 0.51, *p*_*spin*_ < 0.001), indicating a spatial coupling between gene expression and functional alterations (Figure S6a–c). This analysis identified 2097 positively weighted (PLS1+, *Z* > 4.65) and 1881 negatively weighted (PLS1−, *Z* < −4.65) genes significantly associated with the gradient's alteration (*p*_Bonferroni_ < 0.05). The top positively weighted gene was Coiled‐Coil‐Helix‐Coiled‐Coil‐Helix Domain Containing 6 (CHCHD6) (Figure S6d), and the strongest negatively weighted gene was Nuclear Receptor Coactivator 3 (NCOA3) (Figure S6e), with full details in Table S12. A right‐hemisphere sensitivity analysis using available AHBA samples supported the transcriptomic association: Right‐hemisphere PLS1 explained 18.64% of the variance in the regional Gradient‐1 alteration map and showed concordant gene weights with the primary left‐hemisphere analysis.

### 3.6 Transcriptomic Decoding at GAMBA

Transcriptomic decoding of the significant genes (*p*_Bonferroni_ < 1 × 10^−6^) revealed functional and pathological profiles for the PLS1+ and PLS1− sets. The PLS1+ gene set (linked to first gradient disruption) was associated with reduced FC strength (*β* = −0.370) and decreased cerebral metabolism, as reflected by reductions in the cerebral metabolic rate of oxygen (CMRO_2_; *β* = −0.570) and the cerebral metabolic rate of glucose (CMRGlu; *β* = −0.34) (Figure S7; Table S13). Pathologically, this set showed strong positive associations with voxel‐based morphometry (VBM) meta‐analyses of neurodegeneration, most notably dementia (*β* = 0.546), bipolar disorder (*β* = 0.526), FTD (*β* = 0.516) and Alzheimer's disease (*β* = 0.502) (Figure 4). Conversely, the PLS1− gene set was associated with increased FC (*β* = 0.320), elevated metabolism (CMRO_2_; *β* = 0.510) and showed prominent negative VBM associations, including FTD (*β* = −0.524) and bipolar disorder (*β* = −0.514) (Figure 4). These findings indicate distinct spatial associations of the PLS1 gene sets with normative metabolic and neuroimaging annotations.

**FIGURE 4:**
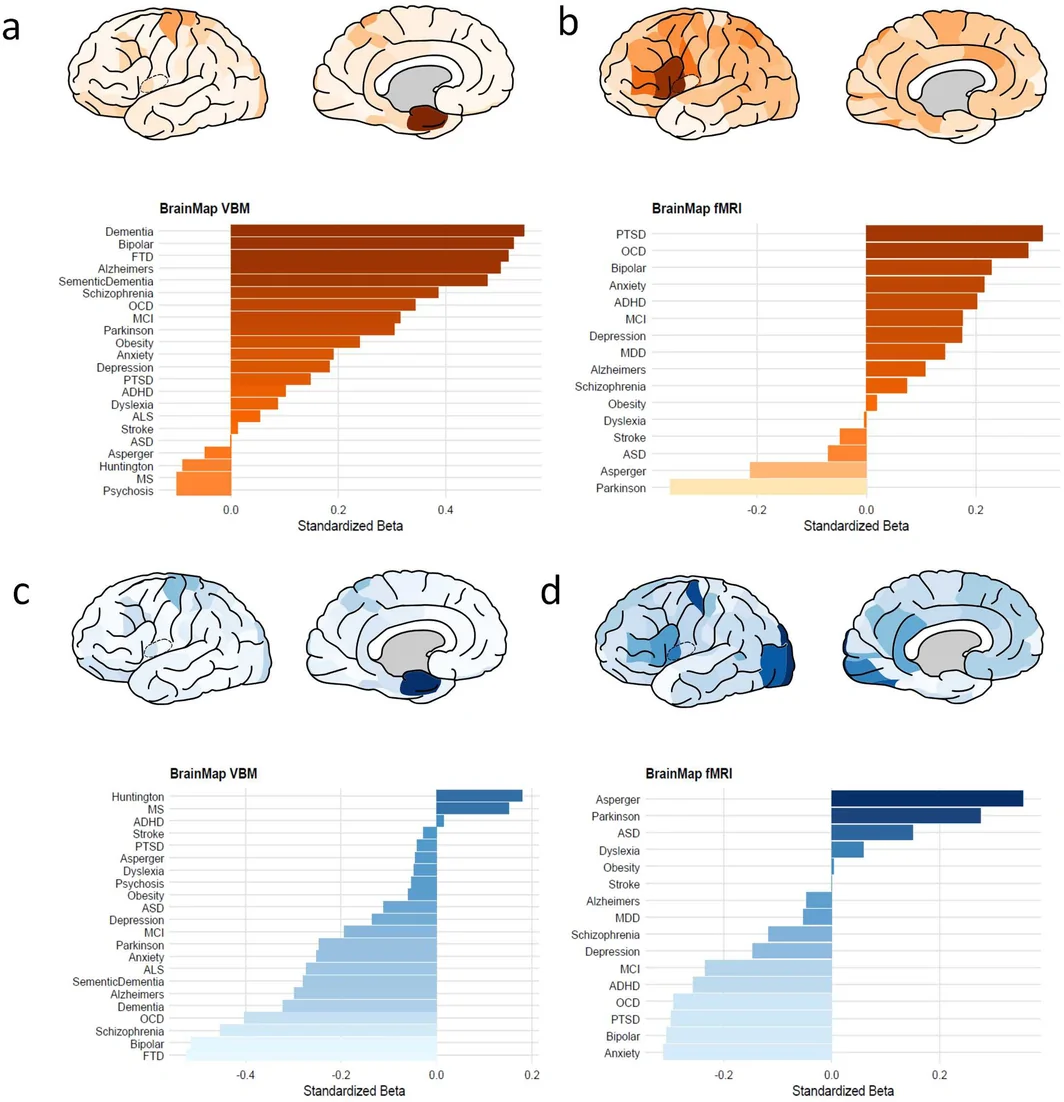
BrainMap decoding of PLS1‐positive and PLS1‐negative gene sets. (a, b) PLS1+ gene‐set associations with (a) voxel‐based morphometry (VBM) and (b) functional MRI meta‐analytic maps. (c, d) Corresponding PLS1− gene‐set associations with (c) VBM and (d) functional MRI meta‐analytic maps. Brain schematics show the spatial distribution of the relevant meta‐analytic phenotype, and adjacent bar plots show standardized regression coefficients (*β*). Positive and negative *β* values indicate positive and negative spatial associations, respectively. Only results meeting the reported multiple‐comparison threshold are displayed.

### 3.7 Gene Set Enrichment and Synaptic GO Annotation

We performed a comprehensive functional annotation to decode the biological architecture of the gradient‐associated genes. For the PLS1+ gene set, GO analysis revealed a significant enrichment in synaptic components (Figure S8a), a finding corroborated by SynGO analysis which mapped 318 genes to specific synaptic structural compartments (Figure S8b). In contrast, the PLS1− gene set was primarily associated with ‘RNA splicing’ and ‘nucleocytoplasmic transport’ (Figure S8c) and notably lacked significant synaptic enrichment after FDR correction (Figure S8d). Detailed functional profiles are provided in Figures S9–S10 and Table S14. To compare these transcriptomic signatures with ALS‐related reference gene sets, we visualised the overlap between the 2091 PLS1+ genes, established ALS risk genes (GeneCards), and 17 causal candidates identified via SMR using a Circos plot (Figure S8e). These overlapping genes formed tightly clustered functional modules based on Kappa similarity (≥ 0.3) (Figure S8f). A parallel overlap analysis was conducted for the PLS1− gene set (Figure S8g), revealing its own distinct clustered enrichment map (Figure S8h). The PLS1+ network highlighted dense interconnections between functional clusters, with a specific hierarchical organisation observed for ‘mitochondrial organisation’. Finally, the PLS1− gene set displayed a distinct network architecture, prominently involving pathways related to ‘muscle structure development’. MCODE analysis further dissected these networks into specific components, such as synaptic signalling and energy metabolism (Figure S11; Table S15).

### 3.8 PLS1‐Gene‐List Enrichment at Cell Type–Specific and Chromosome Levels

To elucidate the biological relevance of the neuroimaging‐derived PLS1 gene lists, we performed cell‐type and chromosome‐level enrichment analyses. Cell‐type enrichment analysis revealed that the top 80% of PLS1+ and PLS1− genes were preferentially expressed in distinct brain cell populations. Integrating results from the AHBA‐seq and DrONc‐seq datasets, PLS1+genes were consistently enriched in oligodendrocytes (ODC). In contrast, PLS1− genes were robustly enriched in astrocytes (ASC) and GABAergic neurons (GABA) (Figure 5a,b). Detailed statistical results are provided in Tables S16 and S17. We further examined the chromosomal distribution of these gene sets using a median rank–based approach (Figure 5c,d). PLS1+ genes were over‐expressed on chromosomes 8 and 1, whereas chromosomes 16 showed significant under‐expression (*p*_perm_ < 0.05). In contrast, PLS1− genes were over‐expressed on chromosomes Y, 16 and 5, whereas chromosomes 12 and X showed significant under‐expression (*p*_perm_ < 0.05), indicating distinct chromosome‐specific expression patterns between the two gene sets. These findings indicate that the gradient‐associated gene sets possess distinct cellular and chromosomal topographies (Table S18).

**FIGURE 5:**
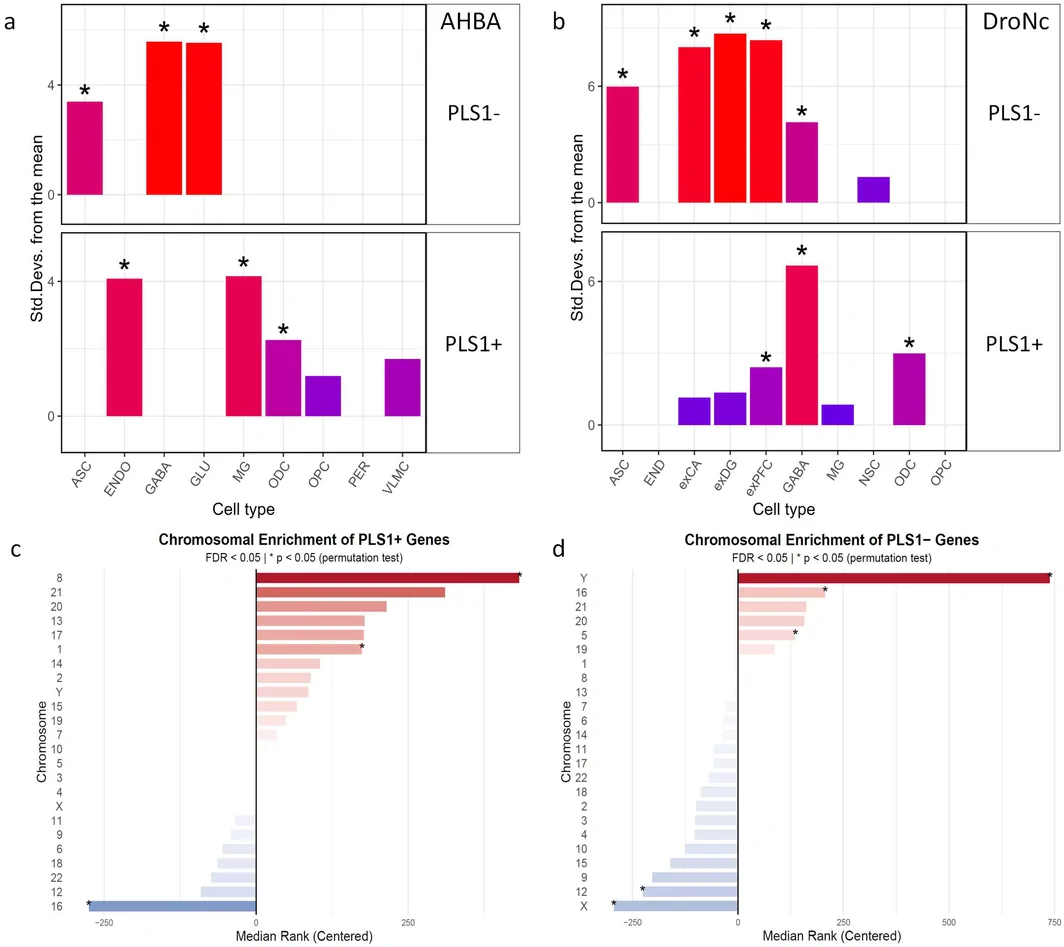
Cell‐type and chromosome‐level enrichment of PLS1 genes. (a) Top 80% PLS1+ and PLS1− genes in AHBA‐seq data; bars plotted only if FDR‐corrected *p* < 0.5, *: FDR‐corrected *p* < 0.05. (b) Top 80% PLS1+ and PLS1− genes in DroNc‐seq data; bars plotted only if FDR‐corrected *p* < 0.5, *: FDR‐corrected *p* < 0.05. (c) Chromosome‐level enrichment of PLS1+ genes across chromosomes 1–22 and XY using a median rank‐based approach. (d) Chromosome‐level enrichment of PLS1− genes across chromosomes 1–22 and XY using a median rank‐based approach.

## 4 Discussion

This study demonstrates significant abnormalities in the brain's functional hierarchical structure in patients with ALS‐ES. Specifically, the gradient scores were found to be increased in the SMN and decreased in the DMN. This result suggests that the functional organisation of the ALS brain undergoes network‐specific reorganisation, leading to a decoupling between previously coordinated functional systems [21]. The increase in SMN gradient values may reflect altered network‐level functional organisation in ALS‐ES [22]. In contrast, lower DMN gradient values may reflect altered organisation of higher order association networks [23], consistent with the common cognitive‐behavioural abnormalities seen in ALS patients, such as impaired executive function and self‐referential processing deficits [24], which can be present even in early stages. This study extends the traditional perspective of ALS as a motor system pathology to the level of whole‐brain functional architecture. It shows that the disease also widely affects cross‐modal systems that support high‐level cognition, thereby providing strong neuroimaging evidence for ALS as a multi‐system neurodegenerative disease.

To uncover the normative neurochemical context of the gradient changes, this study correlated FC gradients with the spatial distribution of 19 neurotransmitters and transporters. The results showed that the 5‐HT4 distribution had the most significant positive correlation with the gradient changes observed in our early‐stage cohort, whereas the NET distribution had the most significant negative correlation. The spatial correspondence with the normative 5‐HT4 map provides a neurochemical topographic context for the Gradient‐1 alteration pattern but does not establish serotonergic involvement in ALS [25]. The negative spatial correspondence with the normative NET map indicates an inverse topographic relationship and should not be interpreted as evidence of locus coeruleus–norepinephrine dysfunction in ALS [26]. Cognitive decoding provided complementary exploratory functional annotation of the imaging pattern. The correspondence with medial prefrontal cortex‐related terms may indicate that regions showing Gradient‐1 alterations overlap with cortical territories commonly implicated in self‐referential and abstract cognitive processes [27]. Likewise, the correspondence with sensorimotor terms may suggest that the imaging pattern extends across regions involved in motor and sensory processing, which have been implicated in ALS‐related functional changes [28, 29]. These topographic associations may help generate hypotheses for future studies combining gradient imaging with targeted neuropsychological and motor assessments; however, they do not demonstrate participant‐level cognitive impairment, noradrenergic abnormalities or motor dysfunction in the present cohort.

Through multidimensional analysis on the GAMBA platform, we found that the PLS1+ and PLS1− gene sets show contrasting effects on brain networks, metabolism and disease associations. High expression of the PLS1+ gene set was negatively correlated with FC strength, as well as the CMRGlu and CMRO_2_. In contrast, high expression of the PLS1− gene set was associated with enhanced FC, indicating an opposing association with functional‐connectivity measures. Furthermore, our analysis of neuropsychiatric disease associations revealed that the PLS1+ gene set was positively correlated with the neuroimaging phenotypes of various disorders, including FTD and bipolar disorder, whereas the PLS1− gene set showed a negative correlation. This result indicates that the transcriptomic features associated with functional gradient dysregulation in ALS have significant molecular co‐variability with multiple neuropsychiatric diseases, indicating spatial co‐variation with neuroimaging meta‐analytic patterns across these conditions, such as synaptic dysfunction, neuroinflammation or energy metabolic imbalance [30]. For instance, the significant enrichment of the PLS1+ gene set in postsynaptic specialisation has also been reported in FTD and bipolar disorder, suggesting overlap with molecular pathways previously reported in neuropsychiatric conditions [31, 32].

Functional enrichment analysis of the PLS1+ and PLS1− gene sets revealed their distinct biological roles. Enrichment analysis of the PLS1+ gene set showed overrepresentation of synaptic‐related terms [33]. This gene set was enriched in synaptic‐related structures and functions, such as ‘postsynaptic specialisation’ and ‘asymmetric synapse’. SynGO analysis further confirmed its central role in synaptic composition and function [34]. This finding suggests a spatial association with the macroscopic brain functional gradient dysregulation to molecular‐level synaptic pathology, supporting a hypothesis that synaptic‐related transcriptional patterns may be relevant to regional vulnerability in ALS [35]. In stark contrast, the PLS1− gene set revealed another unique pathological pathway. This gene set was enriched in gene expression regulation pathways, such as ‘RNA splicing’ and ‘ nucleocytoplasmic transport’. Furthermore, both gene sets converged on core pathways including ‘mitochondrion organisation’ and ‘muscle structure development’, indicating enrichment of pathways also implicated in mitochondrial and muscle‐related biological processes. This molecular connection spanning the central and peripheral nervous systems further underscores the nature of ALS as a multi‐system disease.

Through cell‐type and chromosomal enrichment analysis, we found that the PLS1+ gene set is expressed in ODC. ODC, responsible for forming myelin, is crucial for neuronal electrical signal transmission. In contrast, the PLS1− gene set is primarily enriched in ASC and GABA. GABAergic enrichment provides a cell‐type expression context that may guide future mechanistic studies [36]. Furthermore, chromosome‐level enrichment highlighted ALS‐relevant loci. PLS1–− over‐expression on chromosome 5 and 16 is notable, given that FUS (Chr5) and CHMP2B (Chr16) are established ALS genes involved in RNA metabolism and vesicle trafficking. These findings indicate distinct cell‐type and chromosome‐level distributional patterns across the two gene sets.

This study has several limitations. The modest sample size, female predominance and composition of onset phenotypes in this single‐centre early‐stage cohort may limit generalisability; validation in larger, independent and epidemiologically representative cohorts is required. The cross‐sectional design precludes causal or prognostic inference. Therefore, the observed gradient alterations should not be interpreted as diagnostic or prognostic biomarkers, or as evidence of future functional decline. Although the principal findings remained significant after adjustment for anxiety and depression scores and after exclusion of patients with bulbar onset, residual affective, behavioural and clinical heterogeneity cannot be fully excluded. The transcriptomic analyses used normative AHBA data from six post‐mortem donors, with limited right‐hemisphere coverage, and cannot quantify ALS‐specific gene‐expression changes. Similarly, the normative PET maps provide spatial neurochemical annotations rather than evidence of altered receptor or transporter availability in ALS. PET template resolution and signal‐to‐noise may also influence regional correspondence estimates. The PLS‐derived gene sets are hypothesis‐generating and do not establish therapeutic targets or patient stratification. The cognitive‐decoding results are also hypothesis‐generating and do not establish individual‐level cognitive deficits. Finally, future multimodal studies incorporating white‐matter, spinal‐cord and longitudinal clinical measures may provide a more comprehensive model of ALS.

In conclusion, ALS‐ES showed a collapsed functional hierarchy, with increased somatomotor and decreased DMN gradients. These alterations were spatially associated with normative 5‐HT4 and NET maps and with distinct transcriptomic signatures related to synaptic, metabolic and RNA‐processing pathways. These findings provide a multi‐scale framework for understanding cortical dysfunction in ALS‐ES.

## Funding

This study was supported by the Key R&D Program of Shandong Province (2025CXPT133 and 2022ZLGX03), the Natural Science Foundation of Shandong Province (ZR2023MH180), the Qilu Young Scholarship of Shandong University (20201125), the Shandong Provincial Natural Science Foundation Major Basic Research Project (ZR2023ZD14) and the China Postdoctoral Science Foundation (2025M782111).

## Ethics Statement

This study was approved by the Research Ethics Committee of the School of Medicine, Shandong University. Participant information was collected only after all patients and HCs had been made aware of the purpose of the study and provided their written informed consent.

## Conflicts of Interest

The authors declare no conflicts of interest.

## Supporting information


**Figure S1:** Gradient variance distribution across components. ALS‐ES, early stage amyotrophic lateral sclerosis; HCs, healthy controls.
**Figure S2:** Functional gradient alterations in ALS‐ES patients and HCs. (a) Averaged‐group gradient maps for the first gradient in ALS‐ES patients and HCs. (b) Contraction of the first gradient in ALS‐ES patients and HCs, shown by a histogram. (c) Contraction of the first gradient in ALS‐ES patients, shown by a scatter plot. (d) Averaged‐group gradient maps for the second gradient in ALS‐ES patients and HCs. (e) Contraction of the second gradient in ALS‐ES patients and HCs, shown by a histogram. (f) Contraction of the second gradient in HCs, shown by a scatter plot. ALS‐ES, early stage amyotrophic lateral sclerosis; DMN, default mode network; HCs, healthy controls; SMN, somatomotor network; VIS, visual network.
**Table S1:** Comparison of gradient metrics between ALS‐ES patients and HCs.
**Figure S3:** Ridge plots illustrating the distribution of the first and second gradients across the seven networks. ALS‐ES, early stage amyotrophic lateral sclerosis; DAN, dorsal attention network; DMN, default mode network; FPN, frontoparietal network; HCs, healthy controls; LIM, limbic network; SMN, somatomotor network; VAN, ventral attention network; VIS, visual network.
**Table S2:** Regional differences in the first functional gradient between ALS‐ES and HCs.
**Table S3:** Network differences between ALS‐ES patients and HCs.
**Table S4:** Regional differences in the second functional gradient between ALS‐ES and HCs.
**Table S5:** Clinical correlations of network‐level Gradient‐1 measures.
**TABLE S6:** Group × sex interaction analysis of network‐level Gradient‐1 measures.
**Table S7:** Effect sizes for network‐level Gradient‐1 differences between ALS‐ES patients and healthy controls.
**Table S8:** Sensitivity analyses of network‐level Gradient‐1 differences after adjustment for anxiety and depression symptoms.
**Table S9:** Sensitivity analysis of network‐level Gradient‐1 differences after exclusion of patients with bulbar onset.
**Table S10:** PLS weights and spatial correlations for normative neurochemical PET maps.
**Figure S4:** NeuroSynth cognitive decoding of first functional gradient alterations. (a) Word cloud of terms showing positive spatial correlation, with font size proportional to correlation strength. (b) Word cloud of terms showing negative spatial correlation. (c) Radar plot displaying the top 10 positively correlated cognitive terms. (d) Radar plot displaying the top 10 negatively correlated cognitive terms. dmpfc, dorsomedial prefrontal cortex; m1, primary motor cortex; mpfc, medial prefrontal cortex; si, primary somatosensory cortex; sii, secondary somatosensory cortex.
**Figure S5:** NeuroSynth cognitive decoding of second functional gradient alterations. (a) Word cloud of terms showing positive spatial correlation, with font size proportional to correlation strength. (b) Word cloud of terms showing negative spatial correlation. (c) Radar plot displaying the top 10 positively correlated cognitive terms. (d) Radar plot displaying the top 10 negatively correlated cognitive terms. dmpfc, dorsomedial prefrontal cortex; m1, primary motor cortex; mpfc, medial prefrontal cortex; si, primary somatosensory cortex; sii, secondary somatosensory cortex.
**Table S11:** Top cognitive correlates from NeuroSynth decoding for the first and second functional gradients.
**Figure S6:** Gene expression profiles related to regional variation in the first functional gradient. (a) Cortical distribution of the first functional gradient in the left hemisphere (unthresholded). (b) Weighted gene expression map of regional PLS1 scores in the left hemisphere (unthresholded). (c) A scatterplot shows the relationship between regional PLS1 scores (weighted sum of 15 633 gene expression scores) and regional the first functional gradient values (r = 0.51, pspin < 0.001). (d) Genes strongly positively weighted on PLS1 (e.g., CHCHD6) correlate positively with case–control differences in the first functional gradient (r = 0.44, pspin < 0.001). (e) Genes strongly negatively weighted on PLS1 (e.g., NCOA3) correlate negatively with case–control differences in the first functional gradient (r = −0.37, pspin < 0.001). C8orf46, chromosome 8 open reading frame 46; CHCHD6, coiled‐coil‐helix‐coiled‐coil‐helix domain containing 6; EEPD1, endonuclease/exonuclease/phosphatase family domain containing 1; EPB41, erythrocyte membrane protein band 4.1; IFT22, intraflagellar transport 22; LRRC49, leucine rich repeat containing 49; NCOA3, nuclear receptor coactivator 3; PLS, partial least squares; RASSF4, Ras association domain family member 4; SSTR1, somatostatin receptor 1; TUBB2A, tubulin beta 2A class IIa.
**Table S12:** PLS1‐associated genes ranked by loading significance.
**Figure S7:** GAMBA‐based annotation of PLS1+ and PLS1− gene sets. Panels (a)–(e) show the PLS1+ results and panels (f)–(j) show the PLS1− results. Within each gene set, panels summarise regional expression, cognitive‐component, connectome, metabolic and resting‐state‐network annotations, respectively. Bar plots display standardised regression coefficients; only associations surviving the reported correction are interpreted. Detailed definitions of connectome and metabolic metrics are provided below.
**Table S13:** Network and cognitive associations of PLS components.
**Figure S8:** GO enrichment and synaptic localisation analysis of PLS1 genes. (a–d) Analyses for the PLS1+ gene set. (a) Significantly enriched GO terms in PLS1+ genes. (b) Sunburst plot showing synaptic distribution of PLS1+ genes across structural compartments; colour intensity indicates enrichment significance. (c) Circos plot depicting overlap between PLS1+ genes, an ALS‐related gene set from GeneCards and SMR‐significant genes. (d) Clustered enrichment map of significant GO/KEGG/canonical pathways (clustered by Kappa similarity ≥ 0.3). (e–h) Analyses for the PLS1− gene set. (e) Enriched GO terms associated with RNA splicing and nucleocytoplasmic transport in PLS1− genes. (f) Synaptic distribution of PLS1− genes; no terms remained significant after FDR correction. (g) Gene list overlap for PLS1−, ALS‐related genes and SMR‐significant genes. (h) Clustered enrichment map of PLS1− associated pathways.
**Figure S9:** Gene Ontology and KEGG enrichment analysis for PLS1+ genes. (a–c) Gene‐concept network (cnet) plots illustrating the functional linkages between genes and the significantly enriched GO terms in the (a) biological process, (b) cellular component and (c) molecular function categories. (d) A consolidated bar plot showing the top 10 most significantly enriched terms from each of the three GO categories. (e) Bubble plot visualising the enriched KEGG pathways, with point size and colour representing the number of genes and statistical significance, respectively.
**Table S14:** Detailed Gene Ontology enrichment results for PLS1+ and PLS1− components.
**Figure S11:** Molecular Complex Detection (MCODE) network components for the merged gene lists. (a) MCODE network components derived from the merged GWAS, PLS1+ and GeneCards ALS gene list. (b) MCODE network components derived from the merged GWAS, PLS1− and GeneCards ALS gene list. In both panels, network nodes are displayed as pies, with colours representing the specific contribution of each gene list.
**Table S15:** MCODE components and functional annotations.
**Table S16:** Cell‐type enrichment results using the AHBA‐seq dataset.
**Table S17:** Cell‐type enrichment results using the DroNc‐seq dataset.
**Table S18:** Chromosome enrichment analysis results (median rank approach).

## Data Availability

The data supporting the findings of this study are available within the article and its Supporting Information. Publicly available datasets and resources used in this study were obtained from the following repositories: Human gene expression data are available in the Allen Brain Atlas (https://human.brain‐map.org/static/download), neurotransmitter receptor and transporter data can be obtained from the JuSpace toolbox (https://github.com/juryxy/JuSpace/tree/JuSpace_v1.5/JuSpace_v1.5/PETatlas) and atlas information can be downloaded from the Schaefer‐400 repository (https://github.com/ThomasYeoLab/CBIG). Transcriptomic decoding and functional enrichment analyses were performed using the GAMBA platform (https://dutchconnectomelab.nl/GAMBA/), Metascape (https://metascape.org), SynGO (https://syngoportal.org/) and the GeneCards database (https://www.genecards.org/). All data supporting the findings of this study are provided in this article and the supporting information. Data from the other datasets are not publicly available for download, but access requests can be made to the study investigators.
