# Supplementary material for: Cortical Motor Hierarchy Disruption Is Associated With Functional Status in Early Amyotrophic Lateral Sclerosis
Source: J Cachexia Sarcopenia Muscle. 2026 Oct 25;17(5):e70411. doi: 10.1002/jcsm.70411 (PMC13696156; doi:10.1002/jcsm.70411)
Supplement: Supplementary file 1 [file JCSM-17-e70411-s001.docx]

**Supporting information**

**Cortical Motor Hierarchy Disruption Is Associated with Functional Status in Early Amyotrophic Lateral Sclerosis**

**CONTENT**

**Supplementary Methods**.................................................................................................................1

S1.Study participants.........................................................................................................................1

S2.MRI examination and data preprocessing...................................................................................1

S3.Group comparisons of functional gradients................................................................2

S4.Neurotransmitter and cognitive decoding..................................................................................3

S5.Gene expression processing and transcriptomic association.....................................................4

S6.Transcriptomic and neuropsychiatric decoding via GAMBA....................................................5

S7.Transcriptomic functional enrichment analysis.........................................................................5

S8.Cell-Type and chromosome enrichment analysis......................................................................6

S9.Statistical prioritization of findings............................................................................................7

**Supplementary Results**.............................................................................................................7

S1.Global functional gradient features.............................................................................................7

S2.Regional and network-level functional gradients........................................................................9

S3.Clinical associations and sensitivity analyses .............................................................13

S4.Neurochemical signature for gradient-derived phenotype ..................................................15

S5.Association between cognitive terms and ALS-ES alterations...................................................17

S6.Regional gene expression pattern of gradient perturbation.......................................................20

S7.Transcriptomic decoding at GAMBA........................................................................................23

S8.Gene set enrichment and synaptic gene ontology annotation.....................................................26

S9.Enrichment at cell type-specific and chromosome-levels.................................................33

S10.Statistical prioritization of findings........................................................................................38

**Reference**...............................................................................................................38

**Supplement Methods**

**S1. Study participants**

In the present study, 97 newly diagnosed patients with early-stage ALS between December 2020 and July 2022 were consecutively included. All patients met the Gold Coast criteria^1^. In the present study, similar to our previous studies, ALS patients at King’s stage 1 were defined as early-stage ALS patients; thus, only ALS patients classified as King’s stage 1 were included^2^. The exclusion criteria for ALS patients were as follows: (i) family history of ALS; (ii) inability to complete a magnetic resonance imaging (MRI) scan; (iii) comorbidity of other neurological disorders; and (iv) refusal to participate. In addition, 94 age- and sex-matched healthy controls (HCs) were recruited from the community and were subjected to the same exclusion criteria as the ALS-ES patients. We recorded all patients’ demographic and clinical information, including age, sex, education, family history of neurological disease, comorbid conditions, site of symptom onset, progression rate and disease duration^3^. The revised ALS Functional Rating Scale (ALSFRS-R) was used to assess disease severity^3^. The information was then corroborated by an informant (the patient’s spouse, relative, or primary caregiver). Depression and anxiety were assessed using the Hamilton Depression Rating Scale (HDRS) and Hamilton Anxiety Rating Scale (HARS), respectively^4^.

**S2. MRI examination and data preprocessing**

Structural images of the entire brain were obtained using a 3D fast spoiled gradient-echo sequence with the following parameters: repetition time (TR) = 6.7 ms, echo time (TE) = 3.0 ms, matrix size = 240 × 240 × 170, voxel size = 1 mm × 1 mm × 1 mm, field of view (FOV) = 240 mm × 240 mm, and a total of 180 slices. The rs-fMRI images were obtained using an echo-planar imaging sequence with the following parameters: TR = 2000 ms, TE = 30 ms, FOV = 230 mm × 230 mm × 144 mm, matrix = 68 × 68, voxel size = 3.5 mm × 3.5 mm × 4 mm, slice thickness = 4 mm, no slice gap, and a total of 32 slices and 240 phases. The dcm2niix toolkit was utilized to convert raw DICOM data into the Brain Imaging Data Structure (BIDS) format. Subsequent preprocessing was performed using fMRIPrep 24.1.0rc2. For T1-weighted structural images, processing steps included intensity non-uniformity correction, skull-stripping, and brain tissue segmentation into cerebrospinal fluid, white matter, and gray matter. Brain surfaces were reconstructed using FreeSurfer's recon-all function. Spatial normalization to the ICBM 152 Nonlinear Asymmetrical template (2009c) was applied using nonlinear registration. For functional data, preprocessing included estimation of head-motion parameters, co-registration to the corresponding T1-weighted image using boundary-based registration, and resampling of the BOLD time series onto the fsaverage5 surface. Confound regression was performed using 36 nuisance regressors, comprising motion parameters, global and tissue signals, their temporal derivatives, and quadratic terms. The data were then band-pass filtered (0.01–0.1 Hz) and smoothed on the surface (FWHM = 6 mm). Finally, three global gradient metrics—range, standard deviation, and variance proportion—were calculated to quantify data dispersion and information content. A statistical threshold of *p* < 0.05 was applied for these metrics.

**S3. Group comparisons of functional gradients**

For each canonical network, standardized ALS-HC group differences were quantified using Cohen’s d, calculated as the difference between ALS-ES and HC means divided by the pooled standard deviation. Hedges’g was additionally calculated as a small-sample corrected standardized effect size. Within the ALS-ES group, Pearson correlations were calculated between each network-level Gradient-1 measure and ALSFRS-R score or disease duration. Associations with ALSFRS-R were further examined using multivariable linear regression adjusted for age, sex, mean framewise displacement, HARS, and HDRS. To evaluate the robustness of the primary network-level findings, three sensitivity analyses were conducted. First, Group × sex interaction models were fitted for each canonical network, including Group, sex, the Group × sex interaction term, age, and mean framewise displacement; female participants were used as the reference category. Second, sex-stratified ordinary least-squares models were fitted separately in females and males, adjusted for age and mean framewise displacement. Third, the primary group-comparison models were repeated with HARS and HDRS added as covariates. Benjamini-Hochberg FDR correction was applied across the seven canonical networks separately for each analysis family.

**S4. Neurotransmitter and cognitive decoding**

Multi-site PET data (n=1200) were used to construct a neurotransmitter atlas quantifying 19 receptors/transporters across the Schaefer-400 parcellation^5^. Voxel-wise case-control t-maps of connectome gradients were parcellated to the same atlas. Partial least squares (PLS) regression was applied to fit the neurotransmitter distribution (X: 19×400) to regional gradient differences (Y: 1×400). PLS components were estimated as latent variables (u, v) defined as linear combinations of standardized predictors and responses to maximize covariance. The first component (u1, v1) was obtained via Lagrange multiplier optimization, with vector scores computed from observation matrices (A, B). Regression was iteratively performed to fit Y to u, updating residual matrices (A1, B1) until convergence, with all qualified components extracted when the matrix rank fell below min(n-1, m). Final PLS models captured components with maximal explanatory power. For cognitive decoding, the case-control t-map was converted to volume space in BrainStat^6^, and its spatial correlation with meta-analytic feature maps of cognitive terms from the Neurosynth database was computed. Cognitive decoding was performed by spatially correlating the case-control Gradient-1 t-map with meta-analytic term maps from the Neurosynth database. This analysis was used to generate functional hypotheses regarding the topography of the imaging phenotype; it did not involve participant-level cognitive or neuropsychological measurements and was not intended to infer behavioural impairment. Regional neurotransmitter mapping was performed using a normative multi-tracer PET atlas comprising 19 receptor and transporter maps derived from healthy reference datasets. The case-control Gradient-1 t-map was compared with each parcellated PET map using spatial correlation analysis, and spin-based spatial permutation testing was used to account for cortical spatial autocorrelation. These analyses assess topographic correspondence between the ALS-related imaging phenotype and normative neurochemical distributions; they do not quantify receptor or transporter availability in the ALS-ES participants.

**S5. Gene expression processing and transcriptomic association**

Gene expression data were obtained from the Allen Human Brain Atlas (AHBA), comprising microarray data from six postmortem adult brains (5 males, 1 female; mean age 42.50 ± 13.38 years) covering 3,702 unique anatomical samples^7^. Data processing followed the protocol with the following key steps: (1) probe-to-gene annotations were verified using the Re-annotator toolkit; (2) probes with signal intensities not reliably above background noise in at least 50% of samples were excluded through intensity filtering; (3) for each gene, the probe showing the highest correlation to RNA-seq data was selected; (4) anatomical samples were allocated to regions in the Schaefer-400 parcellation based on a 2 mm Euclidean distance threshold from parcel centroids; (5) gene expression values were normalized within each donor using a scaled robust sigmoid function; and (6) gene set filtering was performed based on differential stability. Due to limited right hemisphere coverage (data from only two donors), analysis was restricted to the left hemisphere, resulting in a final expression matrix of 200 regions × 15,633 genes. For transcriptomic association analysis, partial least squares (PLS) regression was applied to model the relationship between regional gradient alterations (t-values across 200 left-hemisphere regions) and genome-wide expression profiles. The significance of the primary PLS component (PLS1) was assessed using spherical permutation testing (n=1,000) to account for spatial autocorrelation. Gene stability was evaluated through bootstrap resampling, with Z-scores calculated as the ratio of each gene's PLS1 weight to its bootstrap-derived standard error. Genes meeting a Bonferroni-corrected threshold of *p* < 0.05 were considered significantly associated with the gradient alterations. To assess inter-hemispheric robustness, we repeated the PLS analysis using right-hemisphere AHBA expression data and the corresponding 200-region right-hemisphere Gradient-1 alteration map. The same gene-expression preprocessing, PLS-regression framework, random-permutation procedure (1,000 iterations), and bootstrap procedure used in the primary analysis were applied. The significance of the right-hemisphere PLS model was evaluated using random permutations, and concordance between left- and right-hemisphere PLS1 gene weights was quantified using Spearman correlation. Because right-hemisphere AHBA coverage was limited, this analysis was considered a sensitivity analysis rather than an independent replication.

**S6. Transcriptomic and neuropsychiatric decoding via GAMBA**

The GAMBA platform serves as a pathophysiological database for meta-analytic gene-list annotation, facilitating the decoding of gradient relationships and neuropsychiatric associations^8^. Genes identified through a prior PLS analysis and surviving Bonferroni method correction, were bifurcated into positive (PLS1+) and negative (PLS1-) weight subsets. A general linear regression model was then employed to fit these gene sets to various neuroimaging-derived regional and global phenotypes, encompassing resting-state brain networks, cognitive components, cortical metabolism and evolutionary cortical expansion. We used general linear regression model to fit the PLS1+ and PLS1- for the neuroimaging-derived regional/global phenotype, respectively, including resting-state brain network, cognitive brain components, cortical metabolism and evolutionary cortical expansion. Moreover, using activation-likelihood estimate (ALE) analysis, the neuropsychiatric/neurological association of these gene sets was examined at GAMBA. Beta coefficient (β1) for each model was standardized, while the corresponding *p* values were corrected by Bonferroni method at alpha = 0.05.

**S7. Transcriptomic functional enrichment analysis**

We then used all genes in PLS1+ and PLS1- to conduct further bioinformatics analyses investigating whether these genes map to common and relevant biological pathways. First, Gene Ontology (GO) functional annotation was performed on the PLS1 negatively weighted genes using the clusterProfiler package^9^. Subsequently, Metascape online platform (<https://metascape.org>) was used for pathway enrichment analysis of the screened differentially expressed genes (*p*_FDR_ < 0.05)^10^. This study submitted PLS1+ and PLS1- genes to the Synapse Gene Ontology (SynGO) database (<https://syngoportal.org/>) for functional annotation, with cellular component information visualized via a sunburst chart. Furthermore, to evaluate the overlap of these gene sets with established ALS risk factors, we integrated summary data from recent summary-data-based Mendelian randomization (SMR) studies and major genome-wide association studies (GWAS), including a large-scale study involving over 240,000 European individuals (accession: ebi-a-GCST90027164). We subsequently performed SMR analysis using expression quantitative trait loci (eQTL) data from the eQTLGen consortium, BrainMeta, GTEx v8 and methylation QTL (mQTL) data. Causal associations were considered significant under a threshold of *p*_SMR_ < 0.05 and *p*_HEIDI_ > 0.01. Additionally, we compiled a reference list of ALS-associated genes from the GeneCards database^11^. This curated risk gene set served as a comparator in a multi-gene-list enrichment analysis to identify both shared and specific pathways and Gene Ontology terms, with false discovery rate (FDR) correction applied at *p* < 0.05.

**S8. Cell-Type and chromosome enrichment analysis**

This study utilized expression-weighted cell type enrichment (EWCE) to investigate whether PLS1+ and PLS1- gene subset were enriched in specific brain cell types and validated the findings using two independent human-derived cell type-specific gene datasets^12^. We first used single-cell transcriptome data from AHBA-seq^13^ and then repeated the analysis using the DroNc-seq dataset from another study to ensure that the results were not dependent on a single dataset^14^. Previous studies have shown that cell type labels in both datasets are largely consistent, covering astrocytes (ASC), microglia (MG), endothelial cells (ENDO), oligodendrocytes (ODC), oligodendrocyte precursor cells (OPC) and GABAergic neurons (GABA)^15^. To further assess chromosomal enrichment, genes with significant loadings in the PLS1+ and PLS1- gene subset after Bonferroni correction were mapped to chromosomal locations. The median rank method was applied to evaluate whether these genes exhibited non-random clustering along chromosomes. A true median rank was computed for the observed gene set and compared against a null distribution generated through 5000 permutations, each randomly selecting an equal-sized gene set from the full genome. Statistical significance was determined if the true median rank fell below the 5th or above the 95th percentile of the null distribution (*p*_perm_ < 0.05).

**S9. Statistical prioritization of findings**

To improve transparency across the multiple imaging, neurotransmitter, transcriptomic, and enrichment analyses, findings were classified according to their statistical evidential strength. Core findings were defined as prespecified network-level ALS-HC group differences and clinical associations that survived Benjamini–Hochberg FDR correction across the seven canonical networks. Robust secondary findings were defined as results surviving the most stringent correction reported within the relevant analysis family, including Bonferroni correction of PLS-loading statistics across the 19 PET maps, Bonferroni- or FDR-corrected gene-weight and enrichment analyses, corrected GAMBA results, and FDR-corrected EWCE results. Results without across-test correction, or those surviving only nominal thresholds, were classified as exploratory.

**Supplement Results**

**S1. Global functional gradient features**

To enhance interpretability, this study focused on the first two functional gradients, consistent with prior research as they collectively accounted for over 10% of the variance in the overall functional connectivity data (**Supplementary Fig.S1**). In group-level gradient mapping (**Supplementary Fig.S2a**), HCs exhibited stable spatial configurations of unimodal networks - including the visual network (VIS) and SMN - and transmodal networks such as the DMN along the principal gradient. The second gradient revealed a continuous transition from primary visual/somatomotor cortices to the DMN (**Supplementary Fig.S2c**), corroborating the hierarchical transformation from unimodal to transmodal systems. Notably, these topological characteristics remained intact in ALS-ES patients. Quantitative analyses indicated spatial contraction of the first gradient (**Supplementary Fig.S2d, e**) gradients in ALS-ES patients compared to HCs. The first gradient showed significant group differences, with ALS-ES patients exhibiting reduced range (*p* = 0.020), standard deviation (*p* = 0.007) and variance ratio (*p* = 0.008). In contrast, no significant intergroup differences were observed in the second gradient (all *p* > 0.05) (**Supplementary Table S1**), suggesting overall preservation of this dimension of functional architecture in ALS-ES patients.

**Fig. S1 Gradient variance distribution across components.** Abbreviations: ALS-ES, early stage amyotrophic lateral sclerosis; HCs, healthy controls.

**Fig. S2 Functional gradient alterations in ALS-ES patients and HCs.** (a) Averaged-group gradient maps for the first gradient in ALS-ES patients and HCs. (b) Contraction of the first gradient in ALS-ES patients and HCs, shown by a histogram. (c) Contraction of the first gradient in ALS-ES patients, shown by a scatter plot. (d) Averaged-group gradient maps for the second gradient in ALS-ES patients and HCs. (e) Contraction of the second gradient in ALS-ES patients and HCs, shown by a histogram. (f) Contraction of the second gradient in HCs, shown by a scatter plot. **Abbreviations**: ALS-ES, early stage amyotrophic lateral sclerosis; HCs, healthy controls; VIS, visual network; SMN, somatomotor network; DMN, default mode network.

**Table S1. Comparison of gradient metrics between ALS-ES patients and HCs**

| **Metric** | **Gradient** | **ALS-ES** | **HCs** | ***t*** | ***p*** |
| --- | --- | --- | --- | --- | --- |
| Range | first gradient | 0.215 ± 0.030 | 0.225 ± 0.027 | -2.341 | 0.020* |
|  | second gradient | 0.194 ± 0.027 | 0.191 ± 0.033 | 0.562 | 0.575 |
| Standard deviation | first gradient | 0.055 ± 0.009 | 0.059 ± 0.009 | -2.747 | 0.007* |
|  | second gradient | 0.048 ± 0.008 | 0.047 ± 0.009 | 0.480 | 0.632 |
| Variance ratio | first gradient | 0.003 ± 0.001 | 0.004 ± 0.001 | -2.696 | 0.008* |
|  | second gradient | 0.002 ± 0.001 | 0.002 ± 0.001 | 0.278 | 0.781 |

**Abbreviations:** ALS-ES, early stage amyotrophic lateral sclerosis; HCs, healthy controls.

**S2. Regional and network-level functional gradients**

Regression analysis adjusted for age and sex revealed significant alterations in the first functional gradient in ALS-ES patients. At the regional level, ALS-ES patients exhibited decreased in the first-gradient values in DMN nodes, including prefrontal (LH_Default_PFC_1, RH_Default_PFCdPFCm_2/4/5) and temporal regions (LH_Default_Temp_6), alongside increased values in bilateral SMN regions (LH_SomMot_4/9, RH_SomMot_9/11/12/14) (**Supplementary Table S2**). These regional effects were mirrored at the network level, where the DMN showed reduced in the first-gradient values (*t* = -4.124, *p*_FDR_ < 0.001), while the SMN exhibited increased values (*t* = 3.670, *p*_FDR_ = 0.001) (**Supplementary Table S3**). In contrast, no regions showed significant differences in the second gradient (**Supplementary Table S4**). The overall distributions of the first gradient and second gradient across the seven networks are displayed in ridge plots (**Supplementary Fig. S3**). No significant alterations were observed along the second gradient after multiple comparisons correction.

**Fig. S3 Ridge plots illustrating the distribution of the first and second gradients across the seven networks. Abbreviations:** ALS-ES, early stage amyotrophic lateral sclerosis; HCs, healthy controls; VIS, visual network; SMN, somatomotor network; DAN, dorsal attention network; VAN, ventral attention network; LIM, limbic network; FPN, frontoparietal network; DMN, default mode network.

**Table S2. Regional differences in the first functional gradient between ALS-ES and HCs**

| **Region** | **Network** | ***t*** | ***p*_Bonferroni_** |
| --- | --- | --- | --- |
| LH_SomMot_4 | SMN | 4.950 | 0.001 |
| LH_SomMot_9 | SMN | 4.715 | 0.002 |
| LH_Default_PFC_1 | DMN | -4.327 | 0.010 |
| LH_Default_Temp_6 | DMN | -4.817 | 0.001 |
| RH_Default_PFCdPFCm_2 | DMN | -4.478 | 0.005 |
| RH_Default_PFCdPFCm_4 | DMN | -4.160 | 0.019 |
| RH_Default_PFCdPFCm_5 | DMN | -4.989 | 0.001 |
| RH_SomMot_11 | SMN | 5.599 | < 0.001 |
| RH_SomMot_12 | SMN | 4.163 | 0.019 |
| RH_SomMot_14 | SMN | 4.313 | 0.010 |
| RH_SomMot_9 | SMN | 4.369 | 0.008 |

**Abbreviations:** LH, left hemisphere; RH, right hemisphere; SomMot, somatomotor network; SMN, somatomotor network; Default, default mode network; PFC, prefrontal cortex; PFCdPFCm, dorsal and medial prefrontal cortex; Temp, temporal; DMN, default mode network.

**Table S3. Network differences between ALS-ES patients and HCs**

| **Gradient** | **Network** | ***t*** | ***p*** | ***p*_FDR_** |
| --- | --- | --- | --- | --- |
| first | VIS | 2.142 | 0.033 | 0.063 |
| first | SMN | 3.670 | < 0.001 | 0.001 |
| first | DAN | -0.202 | 0.840 | 0.840 |
| first | VAN | 1.066 | 0.288 | 0.403 |
| first | LIM | -2.110 | 0.036 | 0.063 |
| first | FPN | -0.297 | 0.767 | 0.840 |
| first | DMN | -4.124 | < 0.001 | < 0.001 |
| second | VIS | 0.255 | 0.799 | 0.846 |
| second | SMN | 1.144 | 0.254 | 0.846 |
| second | DAN | -0.653 | 0.514 | 0.846 |
| second | VAN | -0.195 | 0.846 | 0.846 |
| second | LIM | -0.376 | 0.707 | 0.846 |
| second | FPN | -1.255 | 0.211 | 0.846 |
| second | DMN | -0.824 | 0.411 | 0.846 |

Abbreviations: VIS, visual network; SMN, somatomotor network; DAN, dorsal attention network; VAN, ventral attention network; LIM, limbic network; FPN, frontoparietal network; DMN, default mode network.

**Table S4. Regional differences in the second functional gradient between ALS-ES and HCs**

| **Region** | **Network** | ***t*** | ***p*** | ***p*_Bonferroni_** |
| --- | --- | --- | --- | --- |
| LH_Vis_8 | VIS | -2.542 | 0.012 | 0.473 |
| LH_SomMot_2 | SMN | -2.776 | 0.006 | 0.346 |
| LH_SomMot_21 | SMN | -2.172 | 0.031 | 0.474 |
| LH_DorsAttn_Post_4 | DAN | -2.221 | 0.028 | 0.474 |
| LH_SalVentAttn_FrOperIns_9 | VAN | 2.295 | 0.023 | 0.474 |
| LH_Limbic_TempPole_6 | LIM | 1.978 | 0.049 | 0.559 |
| LH_Cont_PFCv_1 | FPN | 2.161 | 0.032 | 0.474 |
| LH_Default_Par_6 | DMN | -2.368 | 0.019 | 0.474 |
| LH_Default_PFC_9 | DMN | 2.164 | 0.032 | 0.474 |
| LH_Default_PFC_11 | DMN | 2.328 | 0.021 | 0.474 |
| LH_Default_PFC_12 | DMN | 3.220 | 0.002 | 0.223 |
| LH_Default_PFC_13 | DMN | 2.313 | 0.022 | 0.474 |
| LH_Default_PFC_24 | DMN | 2.414 | 0.017 | 0.474 |
| LH_Default_pCunPCC_1 | DMN | -3.014 | 0.003 | 0.223 |
| LH_Default_pCunPCC_2 | DMN | -3.701 | < 0.001 | 0.113 |
| RH_Vis_16 | VIS | -2.316 | 0.022 | 0.474 |
| RH_DorsAttn_Post_8 | DAN | 2.508 | 0.013 | 0.473 |
| RH_DorsAttn_Post_10 | DAN | 2.236 | 0.027 | 0.474 |
| RH_DorsAttn_Post_17 | DAN | 1.983 | 0.049 | 0.559 |
| RH_Cont_Par_1 | FPN | -2.264 | 0.025 | 0.474 |
| RH_Cont_Par_4 | FPN | 2.287 | 0.023 | 0.474 |
| RH_Cont_PFCl_1 | FPN | -2.973 | 0.003 | 0.223 |
| RH_Default_PFCv_1 | DMN | 2.077 | 0.039 | 0.522 |
| RH_Default_PFCv_2 | DMN | 2.215 | 0.028 | 0.474 |
| RH_Default_PFCv_4 | DMN | 2.183 | 0.030 | 0.474 |
| RH_Default_PFCdPFCm_2 | DMN | 3.154 | 0.002 | 0.223 |
| RH_Default_PFCdPFCm_3 | DMN | 2.099 | 0.037 | 0.513 |
| RH_Default_PFCdPFCm_4 | DMN | 2.626 | 0.009 | 0.428 |
| RH_Default_PFCdPFCm_5 | DMN | 2.447 | 0.015 | 0.474 |
| RH_Default_PFCdPFCm_7 | DMN | 2.001 | 0.047 | 0.559 |
| RH_Default_PFCdPFCm_9 | DMN | 2.615 | 0.010 | 0.428 |
| RH_Default_PFCdPFCm_13 | DMN | 2.109 | 0.036 | 0.513 |
| RH_Default_pCunPCC_1 | DMN | -3.001 | 0.003 | 0.223 |

**Abbreviations:** LH, left hemisphere; RH, right hemisphere; Vis, visual network; VIS, visual network; SomMot, somatomotor network; SMN, somatomotor network; DorsAttn, dorsal attention network; DAN, dorsal attention network; SalVentAttn, salience/ventral attention network; VAN, salience/ventral attention network; FrOperIns, frontal operculum and insula; Limbic, limbic network; LIM, limbic network; TempPole, temporal pole; Cont, control network; FPN, frontoparietal network; PFCv, ventral prefrontal cortex; Default, default mode network; DMN, default mode network; Par, parietal; PFC, prefrontal cortex; pCunPCC, precuneus and posterior cingulate cortex; PFCdPFCm, dorsal and medial prefrontal cortex.

**S3. Clinical associations and sensitivity analyses**

Within the ALS-ES group, higher SMN Gradient-1 values were associated with lower ALSFRS-R scores (*r* = -0.274, *p*_FDR_ = 0.015). Higher dorsal attention network values were associated with lower ALSFRS-R scores (*r* = -0.322, *p*_FDR_ = 0.005), whereas higher DMN values were associated with higher ALSFRS-R scores (*r* = 0.344, *p*_FDR_ = 0.004); no association with disease duration survived FDR correction (**Supplementary Table S5**). In sex-stratified analyses, higher SMN and lower DMN Gradient-1 values remained significant in females (both *p*_FDR_ = 0.0066), while lower DMN values remained significant in males (β = -0.0087, *p*_FDR_ = 0.040). No Group × sex interaction survived FDR correction (all *p*_FDR_ ≥ 0.573; **Supplementary Table S6**). SMN and DMN group differences were moderate in magnitude (Cohen’s d = 0.517 and -0.592, respectively; **Supplementary Table S7**). The primary SMN increase and DMN decrease remained significant after additional adjustment for mean framewise displacement, HARS, and HDRS (SMN: β = 0.0053, *p*_FDR_ = 0.0138; DMN: β = -0.0073, *p*_FDR_ = 0.0138; **Supplementary Table S8**). After exclusion of the 23 patients with bulbar onset, higher SMN Gradient-1 values (β = 0.0040, *p*_FDR_ = 0.0319) and lower DMN Gradient-1 values (β = -0.0077, *p*_FDR_ < 0.001) remained significant in limb-onset ALS-ES patients (**Supplementary Table S9**).

**Table S5. Clinical correlations of network-level Gradient-1 measures**

| **Network** | **ALSFRS-R: r** | ***p*** | ***p*_FDR_** | **Disease duration: r** | ***p*** | ***p*_FDR_** |
| --- | --- | --- | --- | --- | --- | --- |
| VIS | −0.038 | 0.711 | 0.711 | −0.029 | 0.775 | 0.775 |
| SMN | −0.274 | 0.007 | 0.015 | −0.035 | 0.735 | 0.775 |
| DAN | −0.322 | 0.001 | 0.005 | −0.171 | 0.094 | 0.659 |
| VAN | −0.217 | 0.033 | 0.057 | 0.047 | 0.645 | 0.775 |
| LIM | 0.186 | 0.068 | 0.095 | −0.091 | 0.377 | 0.775 |
| FPN | 0.057 | 0.579 | 0.676 | 0.096 | 0.348 | 0.775 |
| DMN | 0.344 | 0.001 | 0.004 | 0.075 | 0.467 | 0.775 |

**Table S6. Group × sex interaction analysis of network-level Gradient-1 measures**

| **Network** | **Beta** | **SE** | ***t*** | ***p*** | ***p*_FDR_** |
| --- | --- | --- | --- | --- | --- |
| VIS | 0.008 | 0.006 | 1.398 | 0.164 | 0.573 |
| SMN | < 0.001 | 0.003 | -0.022 | 0.982 | 0.982 |
| DAN | 0.002 | 0.003 | 0.794 | 0.428 | 0.647 |
| VAN | -0.003 | 0.003 | -0.996 | 0.321 | 0.647 |
| LIM | -0.003 | 0.004 | -0.737 | 0.462 | 0.647 |
| FPN | -0.005 | 0.003 | -1.475 | 0.142 | 0.573 |
| DMN | -0.001 | 0.004 | -0.309 | 0.758 | 0.884 |

**Table S7. Effect sizes for network-level Gradient-1 differences between ALS-ES patients and healthy controls**

| **Network** | **ALS, mean ± SD** | **HC, mean ± SD** | **Cohen’s d** | **Hedges’ g** |
| --- | --- | --- | --- | --- |
| VIS | −0.0533 ± 0.0183 | −0.0596 ± 0.0202 | 0.323 | 0.322 |
| SMN | −0.0308 ± 0.0093 | −0.0359 ± 0.0105 | 0.517 | 0.515 |
| DAN | −0.0242 ± 0.0102 | −0.0240 ± 0.0105 | −0.018 | −0.018 |
| VAN | −0.0155 ± 0.0121 | −0.0169 ± 0.0105 | 0.118 | 0.118 |
| LIM | 0.0241 ± 0.0133 | 0.0282 ± 0.0138 | −0.302 | −0.301 |
| FPN | 0.0270 ± 0.0103 | 0.0276 ± 0.0117 | −0.057 | −0.057 |
| DMN | 0.0597 ± 0.0134 | 0.0673 ± 0.0121 | −0.592 | −0.590 |

**Table S8. Sensitivity analyses of network-level Gradient-1 differences after adjustment for anxiety and depression symptoms**

| **Network** | **Group β** | **SE** | ***p*** | ***p*_FDR_** |
| --- | --- | --- | --- | --- |
| VIS | 0.0075 | 0.0036 | 0.0377 | 0.0660 |
| SMN | 0.0053 | 0.0018 | 0.0040 | 0.0138 |
| DAN | 0.0007 | 0.0019 | 0.7109 | 0.8293 |
| VAN | −0.0002 | 0.0021 | 0.9148 | 0.9148 |
| LIM | −0.0065 | 0.0025 | 0.0099 | 0.0232 |
| FPN | −0.0010 | 0.002 | 0.6186 | 0.8293 |
| DMN | −0.0073 | 0.0023 | 0.0021 | 0.0138 |

**Note:** Models were additionally adjusted for HARS and HDRS, in addition to age, sex, and mean framewise displacement. Benjamini–Hochberg FDR correction was applied across the seven networks.

**Table S9 Sensitivity analysis of network-level Gradient-1 differences after exclusion of patients with bulbar onset**

| **Network** | **Group β** | **SE** | ***t*** | ***p*** | ***p*_FDR_** |
| --- | --- | --- | --- | --- | --- |
| VIS | 0.0076 | 0.0031 | 2.493 | 0.0137 | 0.0319 |
| SMN | 0.004 | 0.0016 | 2.584 | 0.0106 | 0.0319 |
| DAN | -0.0007 | 0.0016 | -0.423 | 0.6727 | 0.7848 |
| VAN | 0.001 | 0.0018 | 0.550 | 0.5830 | 0.7848 |
| LIM | -0.0031 | 0.0021 | -1.493 | 0.1373 | 0.2404 |
| FPN | -0.0001 | 0.0018 | -0.053 | 0.9578 | 0.9578 |
| DMN | -0.0077 | 0.0019 | -4.004 | <0.001 | 0.0007 |

**S4. Neurochemical signature for gradient-derived phenotype**

A Partial Least Squares (PLS) analysis was performed to associate neurotransmitter signatures with initial gradient changes. The importance of the primary component (PLS1) was evaluated through a permutation-based bootstrapping test. The relationship between the PLS1-weighted expression pattern and the gradient differences was assessed through a spatial permutation test. Neurochemical markers were identified according to their Z-score weights. The significance thresholds were established at Z > 2.10 (*p_FDR_* < 0.05) for positive associations and Z < -2.10 (*p_FDR_* < 0.05) for negative associations. The Gradient-1 alteration map showed positive spatial correspondence with the normative 5-HT4 receptor map (*r* = 0.353, *p_spin_* = 0.004) and negative spatial correspondence with the normative NET map. These spatial associations should be interpreted as exploratory normative topographic correspondence. Complete PLS-loading statistics and spatial-correlation coefficients with spin-test P values are reported in **Supplementary Table S10**.

**Table S10. PLS weights and spatial correlations for normative neurochemical PET maps**

| **Neurotransmitter** | ***Z*** | ***p*** | ***r*** | ***p_spin_*** | ***p_Bonferroni_*** | ***p_FDR_*** |
| --- | --- | --- | --- | --- | --- | --- |
| 5HT4 | 7.993 | < 0.001 | 0.353 | 0.004 | < 0.001 | < 0.001 |
| 5HT2a | 7.057 | < 0.001 | 0.373 | 0.002 | < 0.001 | < 0.001 |
| MOR | 6.786 | < 0.001 | 0.365 | 0.003 | < 0.001 | < 0.001 |
| CB1 | 5.535 | < 0.001 | 0.274 | 0.018 | < 0.001 | < 0.001 |
| 5HT1a | 4.130 | < 0.001 | 0.278 | 0.016 | 0.001 | < 0.001 |
| M1 | 2.794 | 0.005 | 0.121 | 0.312 | 0.099 | 0.011 |
| 5HT6 | 2.361 | 0.018 | 0.138 | 0.184 | 0.346 | 0.034 |
| D2 | 1.648 | 0.099 | 0.201 | 0.042 | 1.000 | 0.135 |
| H3 | 1.449 | 0.147 | 0.115 | 0.378 | 1.000 | 0.186 |
| A4B2 | 1.335 | 0.182 | 0.080 | 0.682 | 1.000 | 0.216 |
| mGluR5 | 1.193 | 0.233 | 0.116 | 0.342 | 1.000 | 0.260 |
| D1 | 1.055 | 0.292 | 0.072 | 0.724 | 1.000 | 0.308 |
| 5HT1b | 0.262 | 0.794 | 0.012 | 0.928 | 1.000 | 0.794 |
| NMDA | -2.158 | 0.031 | −0.151 | 0.112 | 0.587 | 0.045 |
| GABAa | -2.299 | 0.021 | −0.171 | 0.068 | 0.408 | 0.034 |
| DAT | -2.337 | 0.019 | −0.135 | 0.196 | 0.369 | 0.034 |
| VAChT | -3.144 | 0.002 | −0.199 | 0.048 | 0.032 | 0.004 |
| 5HTT | -3.263 | 0.001 | −0.289 | 0.006 | 0.021 | 0.003 |
| NET | -3.729 | < 0.001 | −0.314 | 0.004 | 0.004 | 0.001 |

**Abbreviations:** 5HT, serotonin receptor (e.g., 5HT4, 5-HT4 receptor); MOR, μ-opioid receptor; CB1, cannabinoid receptor type 1; M1, muscarinic acetylcholine receptor M1; D1/D2, dopamine receptor D1/D2; H3, histamine receptor H3; A4B2, nicotinic acetylcholine receptor α4β2; mGluR5, metabotropic glutamate receptor 5; NMDA, N-methyl-D-aspartate receptor; GABAa, γ-aminobutyric acid type A receptor; DAT, dopamine active transporter; VAChT, vesicular acetylcholine transporter; 5HTT, serotonin transporter; NET, norepinephrine transporter.

**S5. Association between cognitive terms and ALS-ES alterations**

Meta-analytic cognitive decoding (NeuroSynth) revealed that the case-control t-map of the first functional gradient in early-stage ALS was spatially aligned with higher-order cognitive functions. Specifically, alterations were positively correlated with meta-analytic maps of terms related to the default mode network and self-referential cognition, including medial prefrontal cortex (*r* = 0.36), theory of mind (*r* = 0.36), autobiographical memory (*r* = 0.32), and moral processing (*r* = 0.32) (**Supplementary Fig. S4a, c**). Conversely, negative correlations were observed with terms related to primary sensorimotor and somatosensory functions, including somatosensory cortex (*r* = -0.44), secondary somatosensory cortex (*r* = -0.45), tactile processing (*r* = -0.42), primary motor cortex (*r* = -0.37), and hand movements (r = -0.33) (**Supplementary Fig. S4b, d**). We also performed cognitive decoding on the second functional gradient. This analysis showed a similar directional pattern, with positive correlations for DMN-related terms (e.g., mpfc, *r* = 0.31) and negative correlations for sensorimotor terms (e.g., sensorimotor cortex, *r* = -0.14). However, the strength of these associations was substantially weaker than those observed for the first gradient (**Supplementary Fig. S5**). This indicates that ALS-related increases in gradient values in sensorimotor cortices showed spatial correspondence with meta-analytic sensorimotor terms, a relationship most prominently captured by the first functional gradient. The top 10 positive and negative correlations for both gradients are detailed in **Supplementary Table S11**. The Gradient-1 alteration map showed spatial correspondence with selected cognitive and sensorimotor meta-analytic maps. These associations are reported as exploratory functional annotations of the regional imaging pattern and should not be interpreted as evidence of cognitive impairment or altered behaviour in the ALS-ES participants.

**Fig. S4 NeuroSynth cognitive decoding of first functional gradient alterations.** (a) Word cloud of terms showing positive spatial correlation, with font size proportional to correlation strength. (b) Word cloud of terms showing negative spatial correlation. (c) Radar plot displaying the top 10 positively correlated cognitive terms. (d) Radar plot displaying the top 10 negatively correlated cognitive terms. **Abbreviations:** mpfc, medial prefrontal cortex; dmpfc, dorsomedial prefrontal cortex; si, primary somatosensory cortex; sii, secondary somatosensory cortex; m1, primary motor cortex.

**Fig. S5 NeuroSynth cognitive decoding of second functional gradient alterations.** (a) Word cloud of terms showing positive spatial correlation, with font size proportional to correlation strength. (b) Word cloud of terms showing negative spatial correlation. (c) Radar plot displaying the top 10 positively correlated cognitive terms. (d) Radar plot displaying the top 10 negatively correlated cognitive terms. **Abbreviations:** mpfc, medial prefrontal cortex; dmpfc, dorsomedial prefrontal cortex; si, primary somatosensory cortex; sii, secondary somatosensory cortex; m1, primary motor cortex.

**Table S11. Top cognitive correlates from NeuroSynth decoding for the first and second functional gradients**

| **First gradient item** | **Pearson's r** |  | **Second gradient item** | **Pearson's r** |
| --- | --- | --- | --- | --- |
| cortex mpfc | 0.371 |  | mpfc | 0.308 |
| mpfc | 0.360 |  | cortex mpfc | 0.297 |
| theory mind | 0.360 |  | dorsomedial prefrontal | 0.271 |
| dorsal medial | 0.356 |  | medial pfc | 0.263 |
| dorsomedial prefrontal | 0.350 |  | dorsomedial | 0.262 |
| self referential | 0.348 |  | cortex dmpfc | 0.252 |
| referential | 0.341 |  | medial prefrontal | 0.251 |
| cortex dmpfc | 0.332 |  | dmpfc | 0.244 |
| medial prefrontal | 0.327 |  | dorsal medial | 0.230 |
| mental states | 0.326 |  | moral | 0.192 |
| muscle | -0.388 |  | postcentral gyrus | -0.090 |
| somatosensory cortex | -0.397 |  | motor cortex | -0.095 |
| secondary somatosensory | -0.406 |  | movements | -0.098 |
| primary secondary | -0.412 |  | executed | -0.100 |
| si | -0.412 |  | postcentral | -0.100 |
| touch | -0.413 |  | movement | -0.101 |
| primary somatosensory | -0.414 |  | primary motor | -0.107 |
| tactile | -0.417 |  | touch | -0.107 |
| somatosensory cortices | -0.419 |  | primary sensorimotor | -0.108 |
| somatosensory | -0.444 |  | m1 | -0.108 |
| sii | -0.446 |  | sensorimotor cortex | -0.137 |

**Abbreviations:** mpfc, medial prefrontal cortex; dmpfc, dorsomedial prefrontal cortex; si, primary somatosensory cortex; sii, secondary somatosensory cortex; m1, primary motor cortex.

**S6. Regional gene expression pattern of gradient perturbation**

We used PLS regression to identify the spatial pattern of gene expression that correlated with the anatomical distribution of the first cortical gradient (**Supplementary Fig. S6a**). The PLS1 explained the largest proportion of variance in the first cortical gradient (23.50%) and was significantly above chance (*p_boot_* = 0.001). The distribution of the PLS1 weighted map reflected a robust anterior-posterior gene expression gradient (**Supplementary Fig. S6b**). Notably, the PLS1 weighted gene expression map was spatially correlated with the case-control t-map of the first gradient (*r* = 0.51, *p_spin_* < 0.001) (**Supplementary Fig. S6c**), indicating that positively weighted genes on PLS1 were more highly expressed in cortical regions with increased the first gradient values. Conversely, negatively weighted genes on PLS1 showed lower expression in regions with higher the first gradient scores. Ranking normalized PLS1 weights with univariate one-sample Z tests identified 2097 positively weighted genes (PLS1+, *Z* > 4.65) and 1881 negatively weighted genes (PLS1-, *Z* < -4.65), all surviving Bonferroni correction (*p_bonferroni_* < 0.05). The top positively weighted gene was Coiled-Coil-Helix-Coiled-Coil-Helix Domain Containing 6 (CHCHD6) (**Supplementary Fig. S6d**), while the strongest negatively weighted gene was Nuclear Receptor Coactivator 3 (NCOA3) (**Supplementary Fig. S6e**). The top genes are detailed in **Supplementary Table S12**. In the right-hemisphere sensitivity analysis, PLS1 explained 18.64% of the variance in the regional Gradient-1 alteration map. Right-hemisphere PLS1 scores were positively correlated with the right-hemisphere imaging phenotype (*r* = 0.432, *p* = 1.74×10^-10^), and the empirical permutation *p*-value was 0.001. Left- and right-hemisphere PLS1 gene weights were strongly concordant (Spearman’s ρ = 0.792). These results support the robustness of the principal transcriptomic association across hemispheres, while the limited right-hemisphere donor coverage remains an important limitation. PLS1+ and PLS1- gene sets should be interpreted as hypothesis-generating molecular annotations of the Gradient-1 alteration map. The enrichment of mitochondrial-energy, oligodendrocyte-related, astrocytic, GABAergic, and RNA-processing pathways may help prioritize future patient-derived, longitudinal, and experimental studies, but does not demonstrate therapeutic efficacy or permit patient stratification in the present cohort.

**Fig. S6 Gene expression profiles related to regional variation in the first functional gradient**. (a) Cortical distribution of the first functional gradient in the left hemisphere (unthresholded). (b) Weighted gene expression map of regional PLS1 scores in the left hemisphere (unthresholded). (c) A scatterplot shows the relationship between regional PLS1 scores (weighted sum of 15,633 gene expression scores) and regional the first functional gradient values (*r* = 0.51, *p_spin_* < 0.001). (d) Genes strongly positively weighted on PLS1 (e.g., CHCHD6) correlate positively with case-control differences in the first functional gradient (*r* = 0.44, *p_spin_* < 0.001). (e) Genes strongly negatively weighted on PLS1 (e.g., NCOA3) correlate negatively with case-control differences in the first functional gradient (*r* = -0.37, *p_spin_* < 0.001). **Abbreviations:** C8orf46, chromosome 8 open reading frame 46; CHCHD6, coiled-coil-helix-coiled-coil-helix domain containing 6; EEPD1, endonuclease/exonuclease/ phosphatase family domain containing 1; EPB41, erythrocyte membrane protein band 4.1; IFT22, intraflagellar transport 22; LRRC49, leucine rich repeat containing 49; NCOA3, nuclear receptor coactivator 3; PLS, partial least squares; RASSF4, Ras association domain family member 4; SSTR1, somatostatin receptor 1; TUBB2A, tubulin beta 2A class IIa.

**Table S12. PLS1-associated genes ranked by loading significance**

| **Gene** | **Geneid** | ***Z*** | ***p*** | ***p_Bonferroni_*** | ***p_FDR_*** |
| --- | --- | --- | --- | --- | --- |
| CHCHD6 | 2390 | 14.390 | 5.94E-47 | 9.29E-43 | 4.64E-43 |
| SSTR1 | 12791 | 14.273 | 3.21E-46 | 5.02E-42 | 1.67E-42 |
| IFT22 | 5972 | 14.170 | 1.40E-45 | 2.19E-41 | 5.47E-42 |
| C8orf46 | 1664 | 14.110 | 3.29E-45 | 5.15E-41 | 1.03E-41 |
| TUBB2A | 14206 | 14.009 | 1.37E-44 | 2.14E-40 | 3.57E-41 |
| PPP4R4 | 10274 | 13.987 | 1.87E-44 | 2.93E-40 | 4.18E-41 |
| EFCAB1 | 3770 | 13.911 | 5.44E-44 | 8.51E-40 | 1.06E-40 |
| GDA | 4934 | 13.806 | 2.35E-43 | 3.68E-39 | 4.09E-40 |
| CETN2 | 2337 | 13.780 | 3.36E-43 | 5.26E-39 | 5.26E-40 |
| DTNBP1 | 3640 | 13.741 | 5.76E-43 | 9.01E-39 | 8.19E-40 |
| SECISBP2 | 11738 | -11.336 | 8.68E-30 | 1.36E-25 | 1.64E-27 |
| DIS3L2 | 3413 | -11.352 | 7.26E-30 | 1.13E-25 | 1.40E-27 |
| FASTKD1 | 4432 | -11.383 | 5.05E-30 | 7.90E-26 | 1.01E-27 |
| SYNE4 | 13031 | -11.724 | 9.65E-32 | 1.51E-27 | 2.51E-29 |
| SYCP2 | 13014 | -11.802 | 3.83E-32 | 5.99E-28 | 1.12E-29 |
| RASSF4 | 10874 | -12.171 | 4.46E-34 | 6.97E-30 | 1.70E-31 |
| LRRC49 | 7414 | -12.248 | 1.73E-34 | 2.71E-30 | 7.12E-32 |
| EEPD1 | 3769 | -12.275 | 1.24E-34 | 1.94E-30 | 5.38E-32 |
| EPB41 | 3994 | -12.430 | 1.80E-35 | 2.82E-31 | 8.53E-33 |
| NCOA3 | 8573 | -15.216 | 2.78E-52 | 4.34E-48 | 4.34E-48 |

**Abbreviations:** C8orf46, chromosome 8 open reading frame 46; CETN2, centrin 2; CHCHD6, coiled-coil-helix-coiled-coil-helix domain containing 6; DIS3L2, DIS3 like 3'-5' exoribonuclease 2; DTNBP1, dystrobrevin binding protein 1; EEPD1, endonuclease/exonuclease/phosphatase family domain containing 1; EFCAB1, EF-hand calcium binding domain 1; EPB41, erythrocyte membrane protein band 4.1; FASTKD1, FAST kinase domains 1; GDA, guanine deaminase; IFT22, intraflagellar transport 22; LRRC49, leucine rich repeat containing 49; NCOA3, nuclear receptor coactivator 3; PPP4R4, protein phosphatase 4 regulatory subunit 4; RASSF4, Ras association domain family member 4; SECISBP2, SECIS binding protein 2; SSTR1, somatostatin receptor 1; SYCP2, synaptonemal complex protein 2; SYNE4, spectrin repeat containing nuclear envelope family member 4; TUBB2A, tubulin beta 2A class IIa.

**S7. Transcriptomic decoding at GAMBA**

Using genes with *P*_Bonferroni_ < 1×10^-6^, transcriptomic decoding revealed opposing associations for PLS1+ and PLS1- (**Supplementary Fig. S7, Table. S13**). To annotate cognitive functions, we employed the 12-component ontology system from Yeo et al^16^. We labeled the ontology for each component using the highest probability task, which included: (1) Vibrotactile Monitoring/Discrimination, (2) Recitation/Repetition, (3) Pitch Monitoring/Discrimination, (4) Visual Pursuit/Tracking, (5) Naming, (6) Saccades, (7) Micturition, (8) Flanker Task, (9) Wisconsin Card Sorting Test (WCST), (10) Theory of Mind, (11) Face Monitoring/Discrimination, and (12) Reward Task.

The PLS1+ component was functionally characterized by associations with several cognitive components, reduced functional connectivity (FC) strength (*β* = -0.370) and decreased cerebral metabolism, as reflected by reductions in the cerebral metabolic rate of oxygen (CMRO₂; *β* = -0.570) and the cerebral metabolic rate of glucose (CMRGlu; *β* = -0.34), alongside a negative visual (*β* = -0.580) but positive limbic (*β* = 0.42) profile. This component further showed strong positive pathological associations, particularly in voxel-based morphometry (VBM) meta-analyses with Dementia (*β* = 0.546), Bipolar Disorder (*β* = 0.526), FTD (*β* = 0.516) and Alzheimers Disease (*β* = 0.502) and in functional MRI with Obsessive-Compulsive Disorder (OCD) (*β* = 0.296) and Anxiety (*β* = 0.215).

Conversely, the PLS1- component displayed reversed functional associations including increased FC strength (*β* = 0.320), elevated CMRO_2_ (*β* = 0.510) and a positive visual (*β* = 0.650) but negative limbic (*β* = -0.390) profile (all *P*_FDR_ < 0.05). Pathologically, PLS1- showed prominent negative associations in VBM for FTD (*β* = -0.524), Bipolar Disorder (*β* = -0.514) and Schizophrenia (*β* = -0.454) and in functional MRI for Anxiety (*β* = -0.312) and Post-Traumatic Stress Disorder (PTSD) (*β* = -0.297), suggesting a pattern of disease avoidance or inverse relationship.

**Fig. S7 GAMBA-based annotation of PLS1+ and PLS1− gene sets.** Panels (a-e) show PLS1+ results and panels (f-j) show PLS1- results. Within each gene set, panels summarize regional expression, cognitive-component, connectome, metabolic, and resting-state-network annotations, respectively. Bar plots display standardized regression coefficients; only associations surviving the reported correction are interpreted. Detailed definitions of connectome and metabolic metrics are provided below.

**Table S13. Network and Cognitive Associations of PLS Components**

| **Class** | **Modality** | **Item** | **β** | ***p_boot_*** | ***p_boot_*__adj_** |
| --- | --- | --- | --- | --- | --- |
| PLS+ | RSN | Visual | -0.576 | < 0.001 | 0.003 |
|  | RSN | Somatomotor | -0.295 | < 0.001 | 0.003 |
|  | RSN | Dorsal_attention | -0.070 | 0.275 | 1.000 |
|  | RSN | Ventral_attention | 0.235 | < 0.001 | 0.006 |
|  | RSN | Limbic | 0.415 | < 0.001 | 0.003 |
|  | RSN | Frontal_parietal | 0.167 | 0.005 | 0.133 |
|  | RSN | Default_mode | 0.401 | < 0.001 | 0.003 |
|  | Cognitive_component | Comp01 | -0.229 | < 0.001 | 0.003 |
|  | Cognitive_component | Comp02 | -0.085 | 0.175 | 1.000 |
|  | Cognitive_component | Comp03 | -0.055 | 0.394 | 1.000 |
|  | Cognitive_component | Comp04 | -0.361 | < 0.001 | 0.003 |
|  | Cognitive_component | Comp05 | 0.092 | 0.140 | 1.000 |
|  | Cognitive_component | Comp06 | -0.252 | < 0.001 | 0.003 |
|  | Cognitive_component | Comp07 | 0.073 | 0.245 | 1.000 |
|  | Cognitive_component | Comp08 | 0.328 | < 0.001 | 0.003 |
|  | Cognitive_component | Comp09 | 0.025 | 0.703 | 1.000 |
|  | Cognitive_component | Comp10 | -0.019 | 0.770 | 1.000 |
|  | Cognitive_component | Comp11 | 0.511 | < 0.001 | 0.003 |
|  | Cognitive_component | Comp12 | 0.293 | < 0.001 | 0.003 |
|  | Connectome | Strength_NOS | -0.093 | 0.135 | 1.000 |
|  | Connectome | Strength_FA | 0.023 | 0.717 | 1.000 |
|  | Connectome | Strength_SD | -0.160 | 0.008 | 0.223 |
|  | Connectome | Degree_SC | 0.044 | 0.496 | 1.000 |
|  | Connectome | Strength_FC | -0.370 | < 0.001 | 0.003 |
|  | Metabolic | GI | 0.068 | 0.285 | 1.000 |
|  | Metabolic | OGI | -0.086 | 0.170 | 1.000 |
|  | Metabolic | CMRO2 | -0.570 | < 0.001 | 0.003 |
|  | Metabolic | CMRGlu | -0.340 | < 0.001 | 0.003 |
|  | Metabolic | CBF | -0.170 | 0.004 | 0.128 |
| PLS- | RSN | Visual | 0.651 | < 0.001 | 0.003 |
|  | RSN | Somatomotor | 0.273 | < 0.001 | 0.003 |
|  | RSN | Dorsal_attention | 0.080 | 0.191 | 1.000 |
|  | RSN | Ventral_attention | -0.232 | < 0.001 | 0.006 |
|  | RSN | Limbic | -0.392 | < 0.001 | 0.003 |
|  | RSN | Frontal_parietal | -0.326 | < 0.001 | 0.003 |
|  | RSN | Default_mode | -0.408 | < 0.001 | 0.003 |
|  | Cognitive_component | Comp01 | 0.226 | < 0.001 | 0.009 |
|  | Cognitive_component | Comp02 | 0.102 | 0.089 | 1.000 |
|  | Cognitive_component | Comp03 | -0.029 | 0.641 | 1.000 |
|  | Cognitive_component | Comp04 | 0.486 | < 0.001 | 0.003 |
|  | Cognitive_component | Comp05 | -0.170 | 0.005 | 0.154 |
|  | Cognitive_component | Comp06 | 0.273 | < 0.001 | 0.003 |
|  | Cognitive_component | Comp07 | -0.094 | 0.127 | 1.000 |
|  | Cognitive_component | Comp08 | -0.376 | < 0.001 | 0.003 |
|  | Cognitive_component | Comp09 | -0.094 | 0.123 | 1.000 |
|  | Cognitive_component | Comp10 | 0.115 | 0.060 | 1.000 |
|  | Cognitive_component | Comp11 | -0.459 | < 0.001 | 0.003 |
|  | Cognitive_component | Comp12 | -0.378 | < 0.001 | 0.003 |
|  | Connectome | Strength_NOS | 0.083 | 0.181 | 1.000 |
|  | Connectome | Strength_FA | 0.089 | 0.139 | 1.000 |
|  | Connectome | Strength_SD | 0.180 | 0.003 | 0.075 |
|  | Connectome | Degree_SC | 0.071 | 0.246 | 1.000 |
|  | Connectome | Strength_FC | 0.320 | < 0.001 | 0.003 |
|  | Metabolic | GI | -0.274 | < 0.001 | 0.003 |
|  | Metabolic | OGI | 0.300 | < 0.001 | 0.003 |
|  | Metabolic | CMRO2 | 0.510 | < 0.001 | 0.003 |
|  | Metabolic | CMRGlu | 0.188 | 0.002 | 0.052 |
|  | Metabolic | CBF | 0.064 | 0.293 | 1.000 |

Abbreviations: PLS, partial least squares; RSN, resting-state network. Connectome metrics: Strength_NOS, node strength (number of streamlines); Strength_FA, node strength (fractional anisotropy weighted); Strength_SD, node strength (streamline density weighted); Degree_SC, node degree (structural connectome); Strength_FC, node strength (functional connectome). Metabolic metrics: GI, glycolytic index; OGI, oxygen-glucose index; CMRO_2_, cerebral metabolic rate of oxygen; CMRGlu, cerebral metabolic rate of glucose; CBF, cerebral blood flow.

**S8. Gene set enrichment and synaptic gene ontology annotation**

We performed a comprehensive functional annotation to decode the biological architecture of the gradient-associated genes. For the PLS1+ gene set, Gene Ontology (GO) analysis revealed a significant enrichment in synaptic components (**Fig. S8a**), a finding corroborated by SynGO analysis which mapped 318 genes to specific synaptic structural compartments (**Fig. S8b**). In contrast, the PLS1- gene set was primarily associated with "RNA splicing" and "nucleocytoplasmic transport" (**Fig. S8c**) and notably lacked significant synaptic enrichment after FDR correction (**Fig. S8d**). Detailed functional profiles are provided in **Supplementary Figs. S9-S10 and Table S14**. To bridge these transcriptomic signatures with ALS pathology, we visualized the overlap between the 2097 PLS1+ genes, established ALS risk genes (GeneCards), and 17 causal candidates identified via SMR using a Circos plot (**Fig. S8e**). These overlapping genes formed tightly clustered functional modules based on Kappa similarity (≥ 0.3) (**Fig. S8f**). A parallel overlap analysis was conducted for the PLS1- gene set (**Fig. S8g**), revealing its own distinct clustered enrichment map (**Fig. S8h**). Network visualization further elucidated the functional topology. The PLS1+ network highlighted dense interconnections between functional clusters, with a specific hierarchical organization observed for "mitochondrial organization". Finally, the PLS1- gene set displayed a distinct network architecture prominently involving pathways related to "muscle structure development". MCODE analysis further dissected these networks into specific components, such as synaptic signaling and energy metabolism (**Supplementary Fig. S11, Table S15**).

GO analysis revealed distinct functional profiles for the gradient-associated gene sets (**Supplementary Table S14**). For PLS1+ genes (linked to the first gradient disruption), Biological Process (BP) terms were most significantly enriched in regulation of postsynaptic membrane potential (Z = 7.60), regulation of synaptic plasticity (Z = 7.14), and synaptic vesicle cycle (Z = 6.84). Cellular Component (CC) analysis highlighted neuron-to-neuron synapse (Z = 8.91), asymmetric synapse (Z = 8.51), and postsynaptic specialization (Z = 8.12), while Molecular Function (MF) analysis identified top enrichments in ion channel inhibitor activity (Z = 5.96) and glutamate receptor activity (Z = 5.60) (**Supplementary Fig. S9a-d**). KEGG pathway analysis further identified neuroactive ligand–receptor interaction as the primary pathway (**Supplementary Fig. S9d, e**).

In contrast, for PLS1– genes, BP analysis revealed enrichments in RNA splicing (Z = 5.17), nucleocytoplasmic transport (Z = 4.54), and nuclear export (Z = 4.63). CC terms were dominated by nuclear speck (Z = 4.57), centriole (Z = 4.25), and nuclear periphery (Z = 3.96), and MF analysis showed significant enrichment in histone modifying activity (Z = 5.33), ATP hydrolysis activity (Z = 4.53), and helicase activity (Z = 4.79) (**Supplementary Fig. S10a-d**). KEGG analysis associated these genes with EGFR tyrosine kinase inhibitor resistance (**Supplementary Fig. S10e, f**).

We also performed SynGO analysis using a “brain-expressed” background set (18,035 unique genes, 1,591 overlapping with SynGO). Among PLS1+ genes, 318 out of 2,097 were mapped to SynGO-annotated genes, with 289 and 236 genes assigned to Cellular Component and Biological Process terms, respectively. Notably, 12 CC and 12 BP terms were significantly enriched at 1% FDR. In comparison, only 142 of 1881 PLS1– genes were mapped to SynGO, and while 134 had CC annotations, none showed significant enrichment at 1% FDR, underscoring the specificity of synaptic alterations to the PLS1+ component.

**Fig. S8 GO enrichment and synaptic localization analysis of PLS1 genes.** **(a-d)** Analyses for the PLS1+ gene set. **(a)** Significantly enriched GO terms in PLS1+ genes. **(b)** Sunburst plot showing synaptic distribution of PLS1+ genes across structural compartments; color intensity indicates enrichment significance. **(c)** Circos plot depicting overlap between PLS1+ genes, an ALS-related gene set from GeneCards and SMR-significant genes. **(d)** Clustered enrichment map of significant GO/KEGG/canonical pathways (clustered by Kappa similarity ≥0.3). **(e–h)** Analyses for the PLS1– gene set. **(e)** Enriched GO terms associated with RNA splicing and nucleocytoplasmic transport in PLS1– genes. **(f)** Synaptic distribution of PLS1– genes; no terms remained significant after FDR correction. **(g)** Gene list overlap for PLS1–, ALS-related genes and SMR-significant genes. **(h)** Clustered enrichment map of PLS1– associated pathways.

**Fig. S9 Gene Ontology and KEGG enrichment analysis for PLS1+ genes.** (a-c) Gene-concept network (cnet) plots illustrating the functional linkages between genes and the significantly enriched GO terms in the (a) Biological Process, (b) Cellular Component, and (c) Molecular Function categories. (d) A consolidated bar plot showing the top 10 most significantly enriched terms from each of the three GO categories. (e) Bubble plot visualizing the enriched KEGG pathways, with point size and color representing the number of genes and statistical significance, respectively.

**Fig. S10 Gene Ontology and KEGG enrichment analysis for PLS1- genes.** (a-c) Gene-concept network (cnet) plots illustrating the functional linkages between genes and the significantly enriched GO terms in the (a) Biological Process, (b) Cellular Component, and (c) Molecular Function categories. (d) A consolidated bar plot showing the top 10 most significantly enriched terms from each of the three GO categories. (e) Bubble plot visualizing the enriched KEGG pathways, with point size and color representing the number of genes and statistical significance, respectively.

**Table S14. Detailed Gene Ontology enrichment results for PLS1+ and PLS1- components**

| **Class** | **Ontology** | **ID** | **Description** | **Fold**  **Enrichment** | **zScore** | **qvalue** | **Count** |
| --- | --- | --- | --- | --- | --- | --- | --- |
| PLS1+ | BP | GO:0060078 | regulation of postsynaptic membrane potential | 2.924 | 7.595 | < 0.001 | 41 |
| PLS1+ | BP | GO:0048167 | regulation of synaptic plasticity | 2.496 | 7.143 | < 0.001 | 51 |
| PLS1+ | BP | GO:0042391 | regulation of membrane potential | 1.964 | 6.743 | < 0.001 | 85 |
| PLS1+ | BP | GO:0099003 | vesicle-mediated transport in synapse | 2.337 | 6.920 | < 0.001 | 56 |
| PLS1+ | BP | GO:0099504 | synaptic vesicle cycle | 2.425 | 6.835 | < 0.001 | 50 |
| PLS1+ | CC | GO:0098984 | neuron to neuron synapse | 2.361 | 8.912 | < 0.001 | 90 |
| PLS1+ | CC | GO:0032279 | asymmetric synapse | 2.363 | 8.509 | < 0.001 | 82 |
| PLS1+ | CC | GO:0099572 | postsynaptic specialization | 2.265 | 8.120 | < 0.001 | 83 |
| PLS1+ | CC | GO:0014069 | postsynaptic density | 2.288 | 7.862 | < 0.001 | 76 |
| PLS1+ | CC | GO:0044306 | neuron projection terminus | 3.006 | 7.413 | < 0.001 | 37 |
| PLS1+ | MF | GO:0008200 | ion channel inhibitor activity | 3.733 | 5.963 | 0.001 | 16 |
| PLS1+ | MF | GO:0016248 | channel inhibitor activity | 3.650 | 5.847 | 0.001 | 16 |
| PLS1+ | MF | GO:0141110 | transporter inhibitor activity | 3.571 | 5.735 | 0.001 | 16 |
| PLS1+ | MF | GO:0008066 | glutamate receptor activity | 4.344 | 5.604 | 0.003 | 11 |
| PLS1+ | MF | GO:0030594 | neurotransmitter receptor activity | 2.540 | 4.996 | 0.003 | 24 |
| PLS1- | BP | GO:0008380 | RNA splicing | 1.773 | 5.173 | 0.008 | 71 |
| PLS1- | BP | GO:0006913 | nucleocytoplasmic transport | 1.813 | 4.542 | 0.044 | 51 |
| PLS1- | BP | GO:0051169 | nuclear transport | 1.813 | 4.542 | 0.044 | 51 |
| PLS1- | BP | GO:0051168 | nuclear export | 2.166 | 4.626 | 0.046 | 31 |
| PLS1- | BP | GO:0032092 | positive regulation of protein binding | 3.206 | 4.645 | 0.121 | 13 |
| PLS1- | CC | GO:0016607 | nuclear speck | 1.745 | 4.569 | 0.012 | 59 |
| PLS1- | CC | GO:0005814 | centriole | 2.114 | 4.247 | 0.039 | 28 |
| PLS1- | CC | GO:0034451 | centriolar satellite | 2.229 | 4.131 | 0.045 | 23 |
| PLS1- | CC | GO:0034399 | nuclear periphery | 2.064 | 3.956 | 0.049 | 26 |
| PLS1- | CC | GO:0001650 | fibrillar center | 1.969 | 3.543 | 0.131 | 24 |
| PLS1- | MF | GO:0140993 | histone modifying activity | 1.818 | 5.333 | 0.001 | 69 |
| PLS1- | MF | GO:0016741 | transferase activity, transferring one-carbon groups | 2.083 | 4.932 | 0.004 | 39 |
| PLS1- | MF | GO:0016887 | ATP hydrolysis activity | 1.711 | 4.531 | 0.005 | 62 |
| PLS1- | MF | GO:0004386 | helicase activity | 2.248 | 4.789 | 0.005 | 30 |
| PLS1- | MF | GO:0008168 | methyltransferase activity | 2.026 | 4.551 | 0.007 | 36 |

**Abbreviations:** BP, biological process; CC, cellular component; MF, molecular function; GO, Gene Ontology; PLS1, first partial least squares component.

We integrated our transcriptomic findings with large-scale GWAS and SMR data, identifying 17 genes from our PLS lists with significant causal associations with ALS: C9orf72, G2E3, GGNBP2, GPX3, MOB3B, MOBP, MYO19, RESP18, RPL26L1, SCFD1, SLC9A8, TBK1, TNIP1, TPP1, UNC13A, WHAMM, and ZNHIT3. To explore functional relationships, we constructed a protein-protein interaction (PPI) network and applied Molecular Complex Detection (MCODE) to extract biologically relevant modules (**Supplementary Fig. S11**). This analysis revealed distinct functional clusters for PLS1+ and PLS1- genes (**Supplementary Table S15**). The PLS1+ network showed strong enrichment in modulation of chemical synaptic transmission (logP = -30.9) and synaptic signaling, alongside metabolic sub-clusters related to generation of precursor metabolites and energy (logP = -9.3) and amyloid fiber formation (logP = -7.3). In contrast, the PLS1- network was associated with mRNA metabolic processes (logP = -18.0), chromatin remodeling (logP = -17.5), and neuron projection morphogenesis (logP = -7.1). A shared cluster identified through GeneCards highlighted marked enrichment in muscle system processes (logP = -53.3) and muscle contraction (logP = -48.1).

**Fig. S11 Molecular Complex Detection (MCODE) network components for the merged gene lists.** (a) MCODE network components derived from the merged GWAS, PLS1+ and GeneCards ALS gene list. (b) MCODE network components derived from the merged GWAS, PLS1- and GeneCards ALS gene list. In both panels, network nodes are displayed as pies, with colors representing the specific contribution of each gene list.

**Table S15. MCODE Components and functional annotations**

| Class | Network | Annotation |
| --- | --- | --- |
| PLS1+ analysis | GeneCards | GO:0003012\|muscle system process\|-53.3;GO:0006936\|muscle contraction\|-48.1;GO:0007610\|behavior\|-44.7 |
|  | PLS1+ | GO:0050804\|modulation of chemical synaptic transmission\|-30.9;GO:0099177\|regulation of trans-synaptic signaling\|-30.8;GO:0099536\|synaptic signaling\|-24.2 |
|  | FINAL | GO:0006091\|generation of precursor metabolites and energy\|-9.3;GO:0015980\|energy derivation by oxidation of organic compounds\|-8.8;GO:0006936\|muscle contraction\|-8.6 |
|  | FINAL_MCODE_ALL | GO:0015980\|energy derivation by oxidation of organic compounds\|-7.5;GO:0006091\|generation of precursor metabolites and energy\|-7.1;GO:0016052\|carbohydrate catabolic process\|-7.1 |
|  | FINAL_SUB1_MCODE_2 | R-HSA-977225\|Amyloid fiber formation\|-7.3;GO:0042176\|regulation of protein catabolic process\|-5.7;GO:0051248\|negative regulation of protein metabolic process\|-5.0 |
|  | FINAL_SUB1_MCODE_3 | WP5355\|Metabolic epileptic disorders\|-7.6;GO:0019752\|carboxylic acid metabolic process\|-4.8 |
| PLS1- analysis | GeneCards | GO:0003012\|muscle system process\|-53.3;GO:0006936\|muscle contraction\|-48.1;GO:0007610\|behavior\|-44.7 |
|  | PLS1- | GO:0016071\|mRNA metabolic process\|-18.0;GO:0006338\|chromatin remodeling\|-17.5;GO:0008380\|RNA splicing\|-15.2 |
|  | FINAL | GO:0048812\|neuron projection morphogenesis\|-7.1;GO:0120039\|plasma membrane bounded cell projection morphogenesis\|-7.1;GO:0048858\|cell projection morphogenesis\|-7.0 |

**Abbreviations:** MCODE, Molecular Complex Detection; GO, Gene Ontology; PLS1, first partial least squares component; R-HSA, Reactome pathway; WP, WikiPathways.

**S9. Enrichment at cell type-specific and chromosome-levels**

Cell-type enrichment analysis was performed to assess whether the top 80% of PLS1+ and PLS1- genes associated with the first gradient changes were preferentially expressed in specific brain cell types. Using AHBA-seq data, PLS1+ genes were enriched in endothelial cells (ENDO), microglia (MG), and oligodendrocytes (ODC), whereas PLS1- genes were enriched in astrocytes (ASC), GABAergic neurons (GABA), and glutamatergic neurons (GLU). Detailed statistical results are provided in **Supplementary Tables S16**. In DrONc-seq data: PLS1+ genes were expressed in glutamatergic neurons from the prefrontal cortex (exPFC) and GABA and ODC, while PLS1- genes were expressed in pyramidal neurons from the hippocampal CA region (exCA), granule neurons from the dentate gyrus (exDG), exPFC, GABA and ASC. Detailed statistical results are provided in **Supplementary Tables S17**. Across both datasets, ODC were the common enriched cell type for PLS1+ genes, whereas ASC and GABA were consistently enriched for PLS1- genes. Given the biological complexity of gene expression, we further examined whether the PLS1+ and PLS1- gene lists exhibited chromosome-level enrichment. Using a median rank-based approach, we assessed the enrichment of the neuroimaging-derived PLS1 components across chromosomes 1-22 and XY. PLS1+ genes were over-expressed on chromosome 8 and 1, whereas chromosomes 16 showed significant under-expression (*p*_perm_ < 0.05). In contrast, PLS1- genes were over-expressed on chromosomes Y, 16 and 5, whereas chromosomes 12 and X showed significant under-expression (*p*_perm_ < 0.05), indicating distinct chromosome-specific expression patterns between the two gene sets. Detailed statistical results are provided in **Supplementary Tables S18**.

**Table S16. Cell-type enrichment results using the AHBA-seq dataset.**

| **Class** | **CellType** | **pvalue** | **fold_change** | **SD** | **qvalue** |
| --- | --- | --- | --- | --- | --- |
| PLS1- | GLU | < 0.001 | 1.120 | 5.544 | < 0.001 |
| PLS1- | GABA | < 0.001 | 1.118 | 5.469 | < 0.001 |
| PLS1- | ASC | < 0.001 | 1.132 | 3.418 | < 0.001 |
| PLS1- | OPC | 0.742 | 0.968 | -0.647 | 1.000 |
| PLS1- | PER | 0.921 | 0.916 | -1.354 | 1.000 |
| PLS1- | ENDO | 1.000 | 0.747 | -4.466 | 1.000 |
| PLS1- | MG | 1.000 | 0.733 | -4.562 | 1.000 |
| PLS1- | VLMC | 1.000 | 0.719 | -5.708 | 1.000 |
| PLS1- | ODC | 1.000 | 0.582 | -7.390 | 1.000 |
| PLS1+ | MG | < 0.001 | 1.250 | 4.085 | < 0.001 |
| PLS1+ | ENDO | < 0.001 | 1.243 | 3.951 | < 0.001 |
| PLS1+ | ODC | 0.010 | 1.130 | 2.278 | 0.030 |
| PLS1+ | VLMC | 0.039 | 1.086 | 1.740 | 0.088 |
| PLS1+ | OPC | 0.104 | 1.061 | 1.228 | 0.187 |
| PLS1+ | GABA | 0.656 | 0.991 | -0.422 | 0.984 |
| PLS1+ | PER | 0.976 | 0.875 | -2.011 | 1.000 |
| PLS1+ | ASC | 0.985 | 0.913 | -2.231 | 1.000 |
| PLS1+ | GLU | 1.000 | 0.921 | -3.634 | 1.000 |

**Abbreviations:** PLS1, first partial least squares component; GLU, glutamatergic neurons; GABA, GABAergic neurons; ASC, astrocytes; OPC, oligodendrocyte precursor cells; PER, pericytes; ENDO, endothelial cells; MG, microglia; VLMC, vascular leptomeningeal cells; ODC, oligodendrocytes. SD, standard deviations from the mean; qvalue, false discovery rate adjusted p-value.

**Table S17. Cell-type enrichment results using the DroNc-seq dataset**

| **Class** | **CellType** | **pvalue** | **fold_change** | **SD** | **qvalue** |
| --- | --- | --- | --- | --- | --- |
| PLS1- | exDG | < 0.001 | 1.158 | 9.082 | < 0.001 |
| PLS1- | exPFC | < 0.001 | 1.139 | 8.356 | < 0.001 |
| PLS1- | exCA | < 0.001 | 1.129 | 7.649 | < 0.001 |
| PLS1- | ASC | < 0.001 | 1.123 | 6.089 | < 0.001 |
| PLS1- | GABA | < 0.001 | 1.076 | 4.425 | < 0.001 |
| PLS1- | NSC | 0.116 | 1.036 | 1.235 | 0.193 |
| PLS1- | OPC | 0.849 | 0.979 | -0.973 | 1.000 |
| PLS1- | END | 0.999 | 0.915 | -2.656 | 1.000 |
| PLS1- | MG | 1.000 | 0.752 | -8.540 | 1.000 |
| PLS1- | ODC | 1.000 | 0.691 | -12.947 | 1.000 |
| PLS1+ | GABA | < 0.001 | 1.137 | 7.192 | < 0.001 |
| PLS1+ | ODC | 0.001 | 1.078 | 3.131 | 0.005 |
| PLS1+ | exPFC | 0.012 | 1.043 | 2.363 | 0.040 |
| PLS1+ | exDG | 0.085 | 1.027 | 1.379 | 0.213 |
| PLS1+ | exCA | 0.141 | 1.020 | 1.133 | 0.282 |
| PLS1+ | MG | 0.196 | 1.025 | 0.820 | 0.327 |
| PLS1+ | OPC | 0.628 | 0.993 | -0.339 | 0.897 |
| PLS1+ | ASC | 0.926 | 0.970 | -1.402 | 1.000 |
| PLS1+ | END | 0.999 | 0.907 | -2.956 | 1.000 |
| PLS1+ | NSC | 1.000 | 0.834 | -5.462 | 1.000 |

**Abbreviations:** PLS1, first partial least squares component; exCA, pyramidal neurons of hippocampal CA region; exDG, granule neurons of dentate gyrus; exPFC, glutamatergic neurons of prefrontal cortex; ASC, astrocytes; GABA, GABAergic neurons; NSC, neural stem cells; OPC, oligodendrocyte precursor cells; END, endothelial cells; MG, microglia; ODC, oligodendrocytes; SD, standard deviations from the mean; qvalue, false discovery rate adjusted p-value.

**Table S18. Chromosome enrichment analysis results (Median Rank Approach)**

| **chr** | **group** | **null_lower** | **null_upper** | **median_rank** | **gene_count** | **centered_rank** | **significance** |
| --- | --- | --- | --- | --- | --- | --- | --- |
| 1 | PLS1+ | 871.975 | 1123.500 | 1127.000 | 208.000 | 173.500 | Enriched |
| 1 | PLS1- | 776.975 | 1033.025 | 963.000 | 169.000 | 9.500 | NS |
| 10 | PLS1+ | 804.975 | 1198.025 | 964.000 | 93.000 | 10.500 | NS |
| 10 | PLS1- | 730.925 | 1095.025 | 828.000 | 91.000 | -125.500 | NS |
| 11 | PLS1+ | 815.938 | 1178.013 | 919.000 | 108.000 | -34.500 | NS |
| 11 | PLS1- | 703.925 | 1086.025 | 896.000 | 83.000 | -57.500 | NS |
| 12 | PLS1+ | 817.000 | 1186.050 | 863.000 | 97.000 | -90.500 | NS |
| 12 | PLS1- | 731.975 | 1074.063 | 727.500 | 100.000 | -226.000 | Depleted |
| 13 | PLS1+ | 680.950 | 1354.050 | 1132.000 | 31.000 | 178.500 | NS |
| 13 | PLS1- | 662.988 | 1158.563 | 940.500 | 48.000 | -13.000 | NS |
| 14 | PLS1+ | 768.975 | 1226.000 | 1058.000 | 73.000 | 104.500 | NS |
| 14 | PLS1- | 648.963 | 1141.013 | 916.500 | 48.000 | -37.000 | NS |
| 15 | PLS1+ | 742.375 | 1262.500 | 1020.000 | 58.000 | 66.500 | NS |
| 15 | PLS1- | 713.000 | 1087.000 | 793.000 | 79.000 | -160.500 | NS |
| 16 | PLS1+ | 802.888 | 1199.000 | 679.000 | 96.000 | -274.500 | Depleted |
| 16 | PLS1- | 685.000 | 1125.050 | 1159.000 | 65.000 | 205.500 | Enriched |
| 17 | PLS1+ | 835.963 | 1176.000 | 1130.000 | 118.000 | 176.500 | NS |
| 17 | PLS1- | 718.000 | 1074.138 | 895.500 | 98.000 | -58.000 | NS |
| 18 | PLS1+ | 695.975 | 1338.100 | 890.000 | 33.000 | -63.500 | NS |
| 18 | PLS1- | 601.488 | 1204.063 | 865.000 | 28.000 | -88.500 | NS |
| 19 | PLS1+ | 845.488 | 1159.550 | 1002.500 | 146.000 | 49.000 | NS |
| 19 | PLS1- | 731.975 | 1070.025 | 1040.000 | 109.000 | 86.500 | NS |
| 2 | PLS1+ | 818.975 | 1170.025 | 1043.000 | 117.000 | 89.500 | NS |
| 2 | PLS1- | 738.475 | 1053.513 | 854.000 | 116.000 | -99.500 | NS |
| 20 | PLS1+ | 749.950 | 1238.025 | 1168.000 | 57.000 | 214.500 | NS |
| 20 | PLS1- | 646.313 | 1150.013 | 1110.500 | 42.000 | 157.000 | NS |
| 21 | PLS1+ | 619.963 | 1397.250 | 1264.000 | 20.000 | 310.500 | NS |
| 21 | PLS1- | 558.950 | 1256.738 | 1115.000 | 22.000 | 161.500 | NS |
| 22 | PLS1+ | 755.988 | 1257.588 | 880.000 | 54.000 | -73.500 | NS |
| 22 | PLS1- | 613.950 | 1204.100 | 884.000 | 35.000 | -69.500 | NS |
| 3 | PLS1+ | 834.863 | 1181.013 | 959.000 | 112.000 | 5.500 | NS |
| 3 | PLS1- | 752.950 | 1052.563 | 851.500 | 120.000 | -102.000 | NS |
| 4 | PLS1+ | 798.425 | 1206.563 | 954.000 | 84.000 | 0.500 | NS |
| 4 | PLS1- | 704.000 | 1107.000 | 851.000 | 75.000 | -102.500 | NS |
| 5 | PLS1+ | 803.938 | 1193.013 | 959.000 | 84.000 | 5.500 | NS |
| 5 | PLS1- | 725.438 | 1070.563 | 1089.000 | 98.000 | 135.500 | Enriched |
| 6 | PLS1+ | 814.425 | 1194.038 | 898.500 | 108.000 | -55.000 | NS |
| 6 | PLS1- | 707.925 | 1083.075 | 918.000 | 87.000 | -35.500 | NS |
| 7 | PLS1+ | 802.975 | 1192.525 | 987.500 | 90.000 | 34.000 | NS |
| 7 | PLS1- | 733.975 | 1071.100 | 924.000 | 101.000 | -29.500 | NS |
| 8 | PLS1+ | 752.950 | 1223.075 | 1386.000 | 67.000 | 432.500 | Enriched |
| 8 | PLS1- | 695.975 | 1117.050 | 957.000 | 63.000 | 3.500 | NS |
| 9 | PLS1+ | 787.950 | 1240.000 | 912.000 | 69.000 | -41.500 | NS |
| 9 | PLS1- | 708.000 | 1116.050 | 750.000 | 71.000 | -203.500 | NS |
| X | PLS1+ | 785.975 | 1219.100 | 953.000 | 79.000 | -0.500 | NS |
| X | PLS1- | 675.500 | 1145.000 | 659.500 | 52.000 | -294.000 | Depleted |
| Y | PLS1+ | 48.000 | 1961.050 | 1039.000 | 1.000 | 85.500 | NS |
| Y | PLS1- | 281.975 | 1560.025 | 1692.000 | 5.000 | 738.500 | Enriched |

**S10. Statistical prioritization of findings**

The core findings comprised higher SMN and lower DMN Gradient-1 values in ALS-ES patients, as well as FDR-corrected associations between network-level Gradient-1 measures and ALSFRS-R. The right-hemisphere AHBA-PLS analysis supported the hemispheric robustness of the primary transcriptomic association. Among neurotransmitter maps, several markers showed FDR- or Bonferroni-significant PLS weights. Their spatial correlations with the Gradient-1 alteration map are reported as exploratory normative topographic associations. Cognitive decoding and chromosome-level analyses were retained as exploratory results and were not interpreted as confirmatory evidence.

**Reference**

1.	Shen D, Yang X, Wang Y, et al. The Gold Coast criteria increases the diagnostic sensitivity for amyotrophic lateral sclerosis in a Chinese population. Translational neurodegeneration 2021;10:28.

2.	Shefner JM, Al-Chalabi A, Baker MR, et al. A proposal for new diagnostic criteria for ALS. Clinical neurophysiology : official journal of the International Federation of Clinical Neurophysiology 2020;131:1975-1978.

3.	Cui B, Cui L, Gao J, et al. Cognitive Impairment in Chinese Patients with Sporadic Amyotrophic Lateral Sclerosis. PloS one 2015;10:e0137921.

4.	Liu S, Zhao Y, Ren Q, et al. Amygdala abnormalities across disease stages in patients with sporadic amyotrophic lateral sclerosis. Human brain mapping 2022;43:5421-5431.

5.	Hansen JY, Shafiei G, Markello RD, et al. Mapping neurotransmitter systems to the structural and functional organization of the human neocortex. Nature neuroscience 2022;25:1569-1581.

6.	Larivière S, Bayrak Ş, Vos de Wael R, et al. BrainStat: A toolbox for brain-wide statistics and multimodal feature associations. NeuroImage 2023;266:119807.

7.	Hawrylycz MJ, Lein ES, Guillozet-Bongaarts AL, et al. An anatomically comprehensive atlas of the adult human brain transcriptome. Nature 2012;489:391-399.

8.	Wei Y, de Lange SC, Pijnenburg R, et al. Statistical testing in transcriptomic-neuroimaging studies: A how-to and evaluation of methods assessing spatial and gene specificity. Human brain mapping 2022;43:885-901.

9.	Yu G, Wang LG, Han Y, He QY. clusterProfiler: an R package for comparing biological themes among gene clusters. Omics : a journal of integrative biology 2012;16:284-287.

10.	Zhou Y, Zhou B, Pache L, et al. Metascape provides a biologist-oriented resource for the analysis of systems-level datasets. Nature communications 2019;10:1523.

11.	Safran M, Dalah I, Alexander J, et al. GeneCards Version 3: the human gene integrator. Database : the journal of biological databases and curation 2010;2010:baq020.

12.	Skene NG, Grant SG. Identification of Vulnerable Cell Types in Major Brain Disorders Using Single Cell Transcriptomes and Expression Weighted Cell Type Enrichment. Frontiers in neuroscience 2016;10:16.

13.	Hawrylycz M, Miller JA, Menon V, et al. Canonical genetic signatures of the adult human brain. Nature neuroscience 2015;18:1832-1844.

14.	Habib N, Avraham-Davidi I, Basu A, et al. Massively parallel single-nucleus RNA-seq with DroNc-seq. Nature methods 2017;14:955-958.

15.	Hodge RD, Bakken TE, Miller JA, et al. Conserved cell types with divergent features in human versus mouse cortex. Nature 2019;573:61-68.
